# The effect of neighbouring group participation and possible long range remote group participation in *O-*glycosylation

**DOI:** 10.3762/bjoc.21.27

**Published:** 2025-02-17

**Authors:** Rituparna Das, Balaram Mukhopadhyay

**Affiliations:** 1 SWEET Lab, Department of Chemical Sciences, Indian Institute of Science Education and Research (IISER) Kolkata, Mohanpur, Nadia 741246, India

**Keywords:** chemical *O*-glycosylation, neighbouring group participation, remote group participation, solvent effect, stereocontrol

## Abstract

Stereoselective glycosylations are one of the most challenging tasks of synthetic glycochemists. The protecting building blocks on the glycosides contribute significantly in attaining the required stereochemistry of the resulting glycosides. Strategic installation of suitable protecting groups in the C-2 position, vicinal to the anomeric carbon, renders neighbouring group participation, whereas protecting groups in the distal C-3, C-4, and C-6 positions are often claimed to exhibit remote group participation with the anomeric carbon. Neighbouring group participation and remote group participation are being widely studied to help the glycochemists design the synthetic protocols for multistep synthesis of complex oligosaccharides and in turn, standardise the process of the glycosylation towards a particular stereochemical output. While neighbouring group participation has been quite effective in achieving the required stereochemistry of the produced glycosides, remote participation exhibits comparatively less efficacy in achieving complete stereoselectivity in the glycosylation reactions. Remote participation is a still highly debated topic in the scientific community. However, implementing the participating role of the remote groups in glycosylation reactions is widely practised to achieve better stereocontrol and to facilitate the formation of synthetically challenging glycosidic linkages.

## Introduction

Cell surface glycans in living cells have significantly spurred the scientific curiosity of researchers over the past few decades [1]. Intensive studies revealed that this subtle code of cell surface glycans is extensively responsible for monitoring a plethora of diverse biological phenomena like post-translational modification of proteins [2–3], blood group specificity, fertilisation, embryogenesis and cell growth [4–6]. Moreover, the glycoprotein and glycolipid structures are also capable of eliciting immune and humoral responses in living bodies [7–8]. They also act as information carriers and participate in various innate life functions [9] like cell–cell signalling and recognition [10], adhesion and tumour metastasis [11]. Thus, proper and elaborate studies of carbohydrates became pertinent in order to decipher the various hidden mysteries of life. As a result, glycochemists and glycobiologists in the post-genomic era exhibited a huge interest in the study of ‘Glycomics’ or ‘Carbohydrate Chemistry.’ These truly elevated the recognition of carbohydrates from being mere building blocks to a frontier research topic towards the development of drugs and pharmaceuticals [12–13], diagnostic tools [14], artificial sweeteners [15], cosmetics, and detergents.

Carbohydrates are the most abundant and versatile renewable sources of energy on earth. However, extraction of naturally occurring complex glycans from natural sources is a laborious and cumbersome task. Moreover, the isolated samples provide a low-yield heterogenous mixture of samples with medium to low reproducibility. The presence of even a small fraction of such contaminants in the naturally isolated oligosaccharide sample is highly detrimental where the outcome of the scientific experiments performed with them are concerned [16]. Impurities also invariably make the characterisation of the isolated samples significantly arduous and faulty. Therefore, the syntheses of these important carbohydrates through chemical means became inevitable to elucidate the structure unambiguously and to get hold of their biological implications with minimal doubt [17].

The oligosaccharide repeating units on the exposed cell surface of living organisms portray the combination of monosaccharides bound to each other by covalent glycosidic bonds created by a process known as glycosylation. These repeating units further remain covalently bound to the specific macromolecules in the cell surface, usually proteins or lipids by the process of glycosylation producing glycoproteins or glycolipids, respectively. Nature executes these processes by enzymatic pathways [18] and is often flawless in its desired output. But the scarcity and the cumbersome purification of natural enzymes limit the use of enzymatic protocols and thus, glycoscientists mostly rely on chemical glycosylation to achieve complex oligosaccharide targets. However, chemical glycosylation remains to be a highly complex, ubiquitous and non-templated process for the synthetic chemists [19]. The inherent structural complexity of the carbohydrates and the abundance of free hydroxy groups create more challenges in performing the site-specific chemical glycosylation processes.

## Review

### Principle of glycosylation

**Scheme 1:**
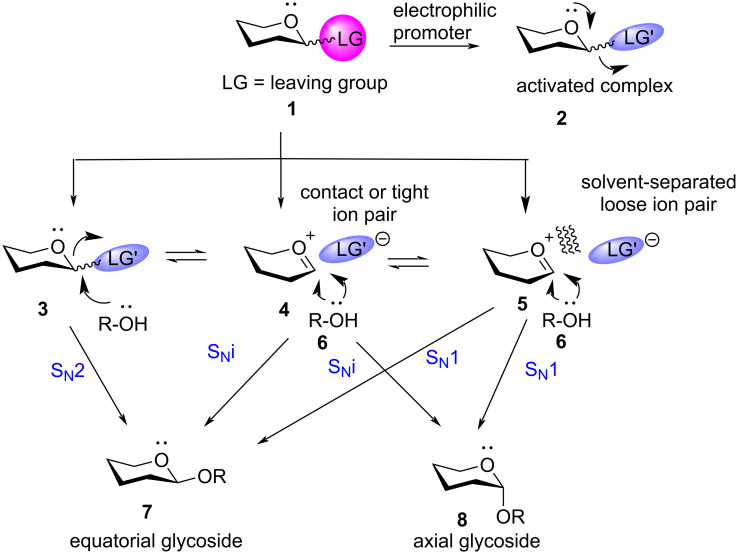
Continuum in the mechanistic pathway of glycosylation [32] reactions ranging between S_N_2 and S_N_1.

The process of formation of a glycosidic bond between two carbohydrate units or a carbohydrate unit with an aglycon is termed as glycosylation. Conventional glycosylation involves the ‘nucleophilic substitution’ of the leaving group at the sp^3^ anomeric centre of the donor moiety with a suitable carbohydrate or non-carbohydrate-based aglycon with the help of an electrophilic promoter to form the equatorial glycoside **7** or the axial glycoside **8** (Scheme 1). Glycosylation is considered as the most crucial step in any oligosaccharide synthesis, although it may be argued that, building block preparations or final deprotection steps remain equally demanding. The main challenge of glycosylation lies in the structural complexity of the carbohydrate moieties [20–22] coupled with the need for suitable anomeric stereoselectivity [23]. Over the decades, there have been widespread studies to standardise and optimise glycosylation reactions, but a standard reaction protocol still eludes the scientific community. We will give a generalised idea of glycosylation mechanisms to enable the readers to have a background idea as a reference before going to describe the role of protecting groups.

The mechanistic pathway of glycosylation strongly depends on many factors, especially, the concentration of the participating moieties, the reaction temperature or the activation entropy, hydrogen bonding, solvent [24], nature of the leaving groups and the promoter used [25]. The mechanism of glycosylation reactions has long been categorised mostly as dissociative S_N_1 reactions proceeding through stabilised oxocarbenium ions with the role of counterions ignored [26–28]. However, recent experimental, kinetic, and physical data reveal the incidence of more associative mechanisms [29–31] wherein the mechanistic pathway of glycosylation seems to lie at an interface of S_N_1–S_N_2 reaction (Scheme 1) [29]. The continuum mechanism expands in two directions away from the central oxocarbenium ion intermediate in the limiting dissociative process involving diastereomeric ion pairs. Destabilisation and greater reactivity of the oxocarbenium intermediate causes the nucleophilic acceptor moiety to attack in a concerted process following a classical S_N_2 pathway through an associative transition state to form the equatorial glycoside, **7**. Surprisingly, recent evidences show that typical homogeneous glycosylation reactions in organic solution shift more towards the S_N_2 end of the mechanistic spectrum [32–34], with some exceptions [35]. The kinetic evidence of the associative end of the mechanistic spectrum has been shown and supported by Crich et al. [36]. The group has also reported instances wherein the triflate counterion may also act as a strong nucleophile [37] to form a loosely bound covalent glycosyl triflate intermediate further leading to the inversion at the anomeric sp^3^ carbon centre by the attack of the acceptor moiety. Crich β-mannosylations are classic illustrations for the same [38–39].

On the other hand, the stability of the carbocation contributes towards the reaction to proceed via the dissociative two-step S_N_1 reaction pathway. The benchmark for the S_N_1 spectrum of the mechanistic continuum has been elaborately illustrated and supported by Codee et al. by mapping full ensemble of conformations that the glycosyl oxocarbenium ions can adopt by a complete conformational energy landscape (CEL) study in a quantitative manner [40]. In such a case, the acceptor has the advantage of attacking from both the sides, and thus, a diastereomeric mixture of both equatorial (**7**) and axial (**8**) anomers are formed [25]. Apart from these, some cases of glycosylation reactions have also been reported to reside along a continuum between the extremes of S_N_1 and S_N_2 pathway by employing the effect of counter ions, showing close resemblance with the S_N_i mechanism [41], conforming to the famous Winstein’s ion-pair theory [42] and draws analogy with chlorination of alcohols by thionyl chloride [43]. Incorporating the role of counter ion pairs in glycosylation mechanisms was first reported by Rhind-Tutt and Vernon [44], and later reiterated by various authors, including the seminal graphical analysis of Lemieux and co-workers [45–47]. Thus, complete categorisation of the reaction in either of the subdomains of substitution reaction is not only difficult but is also defective in principle. This dynamic continuum has also been depicted by an elaborate computational simulation report by Fu et al. in 2021 [41] which was a major follow-up of the work by Crich and co-workers in 2018 [29]. In general cases, the axial glycosides also referred to as α-glycosides are mostly also termed as 1,2-*cis* glycosides (except in the case of glycosides such as ᴅ-altrose, ᴅ-mannose, ᴅ-iodose, and ᴅ-talose with axial C-2 position), while the equatorial or β-glycosides are termed as 1,2-*trans*-glycosides with the glycosides mentioned above as exceptions [48]. The review will primarily be referring to axial or α-glycosides as 1,2-*cis* glycosides except for mannosides where the term β-mannosides will be used for 1,2-*cis*-mannoside configuration, respecting the complexity and novelty they bring to the world of synthetic glycochemistry.

The spanning of the glycosylation reaction between the two extremes of substitution reactions enables the synthetic chemists to manoeuvre and design the coupling reactions according to the regio- and stereochemical demand.

In chemical glycosylation, the role of protecting groups in directing the attack of the incoming nucleophile to obtain the required stereoselective glycosylation is undeniably the most significant factor [49–52]. The role of participating and non-participating protecting groups shows contribution in shifting the S_N_1/S_N_2 spectrum interface of glycosylation, enabling the researchers to utilise them in attaining the required stereospecific glycosylation outputs.

Apart from the widely convenient stepwise synthesis, recently one-pot glycosylations have also made an important mark which minimise the tedious purification of the intermediate molecules in each step [53–58]. In the rigorous planning of one-pot glycosylation the role of a neighbouring group and possible effect of remote participating groups comes into play. One-pot glycosylations have also opened the door of the automated oligosaccharide synthesis which applies the principle of solid-phase synthesis [59–62] and corresponding automated oligosaccharide synthesis [63–65]. These procedures pave the way for the new generation of glycomics and glycochemistry which promises to show new paths towards deciphering the unsolved mysteries of nature [66].

There have been many reviews in literature showcasing the intricate details of glycosylation reactions [67–68] and different protecting groups employed in glycosylation [69]. In this review, we have compiled and critically analysed the contribution of protecting groups in glycosylation reactions both from the perspective of neighbouring group in the vicinal position as well as in a distal or remote position in the glycosyl donors, particularly concentrating on the works in the present millennium post 2000. Involvement of protecting groups in the near, proximal or vicinal position on the stereochemistry of the glycosylation reaction is termed as ‘neighbouring group participation (NGP)’ [70], while involvement of protecting groups in the far, distal or remote position in the glycosyl donor is often termed as ‘remote group participation’ or ‘long distance participation’ [71]. While neighbouring group participation is a much established reaction pathway, there are various studies both supporting and refuting the concept of long-distance participation of building blocks. We will first give an overview of how neighbouring group participation of different protecting groups affects the stereochemistry of the glycoside bonds followed by the role of the same protecting groups as remote groups and analyse the various critical viewpoints pertaining to their distal contribution in determining the stereoselectivity of the glycosylation reactions. The comprehensive knowledge of the role of the protecting groups in glycoside reactions in the different positions depicted in the review may assist the readers to plan their synthetic protocols in oligosaccharide synthesis.

### Neighbouring group participation

In glycosylation reactions the protecting group at the vicinal C-2 position may be either participating or non-participating in nature. Participating functional groups interact with the anomeric carbon and thereby help in the formation of a specific stereocentre. So, modulating the neighbouring protecting group in the C-2 position of glycosyl donors helps in improving the stereoselectivity of the produced glycoside bonds.

#### Ester-type participating protecting groups

Directing from glycosylation with glycosyl halide donors by Fischer [72] and Michael [73] and later modified by Koenigs and Knorr [74], there have been reports of the synthesis of a wide range of glycosides involving the Walden-type inversion in the anomeric position. This concept of Walden inversion in carbohydrates was illustrated extensively by Frush and Isbell [75] with silver carbonate as the promoter, drawing analogy with an S_N_2-type substitution mechanism with the formation of the acetoxonium ion intermediate [76].

**Scheme 2:**
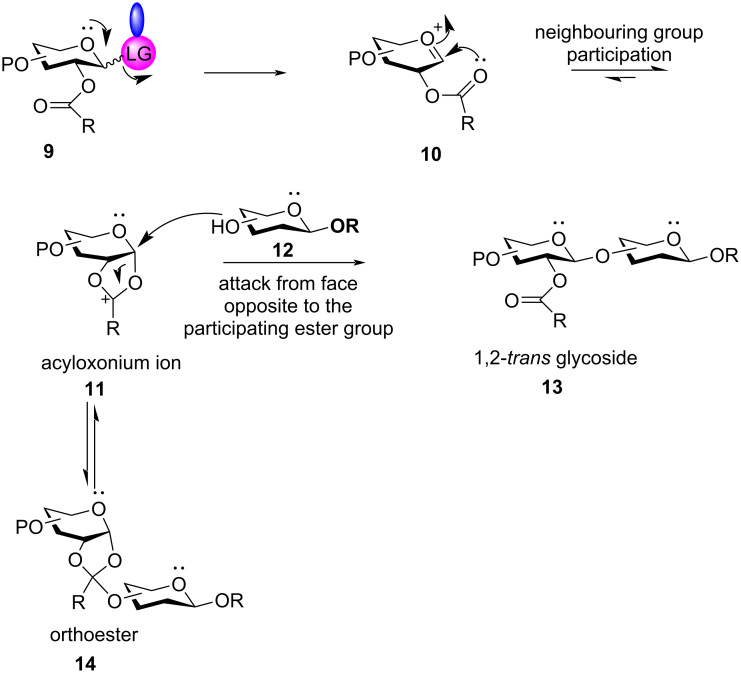
Formation of 1,2-*trans* glycosides by neighbouring group participation with acyl protection in C-2 position.

An acyl protecting group in the vicinal C-2 position is widely accepted as the participating group facilitating the formation of a 1,2-*trans* glycoside (Scheme 2). In general, the cleavage of the activated anomeric leaving group of the glycosyl donor **9** leads to the formation of an electron-deficient oxocarbenium ion **10**. The participating vicinal acyl group interacts with the anomeric carbon forming an electron-deficient bicyclic acyloxonium ion intermediate **11** which blocks the α-face of the glycosyl ring, inducing the attack of the nucleophilic acceptor **12** to approach from the opposite face to form the 1,2-*trans* glycoside **13** primarily.

Various protecting groups for the neighbouring C-2 position have been devised, formulated and standardised providing the neighbouring group assistance to form 1,2-*trans* glycosides [77–78]. However, acyl groups with electron-withdrawing properties in the neighbouring position have been observed to be disarming in nature in most of the cases (exception depicted for perbenzoylated SBox glycosides exhibiting superarmament [79] proving to be more reactive than the analogous perbenzylated donors) thereby reducing the reactivity of the glycosyl donors. The challenge is to activate the ester-protected glycosyl donor and to implement it to obtain significant stereoselective glycosylation products in good yields.

**Acetyl and benzoyl protection:** The total synthesis of oligosaccharides has seen numerous illustrations of the participating role of the acyl esters. Citing a few references in order to elaborate the mechanistic protocol will do injustice to all the numerous other works with acetyl groups. An ester group similar to the acetyl building block, i.e., the benzoyl group, has also been extensively used for the same purpose. Although the use of a benzoyl group in comparison to the acetyl group deviates from the principle of atom economy, it significantly reduces the chance of migration as observed for acetyl groups. Various studies have revealed its dependence on both electron-withdrawing substituents and steric bulk on the carbonyl group [80]. It demonstrates that its migration rate is significantly increased by more electron-withdrawing substituents which also increase the electrophilicity of the carbonyl group. Again, more steric bulk in proximity to the carbonyl group, like in the benzoyl substituent, significantly reduces its migration property [81–82]. Hence, an increasing number of glycochemists across the world successfully substituted the acetyl group with the benzoyl group to primarily obtain 1,2-*trans* glycosides [83–85]. Ishida et al. implemented benzoyl protection for the synthesis of 1,2-*trans* glycosides for the iterative synthesis of oligo-α-rhamnoside derivatives [86]. Both acetyl and benzoyl groups, however, are used synonymously owing to their same participating property and the same Zemplén deprotection strategy [87]. A recent study showed the use of modified Zemplén conditions to synthesise deacetylated methyl β-glycopyranosides directly from per-*O*-acetylated α-glycosyl halides using a stoichiometric amount of sodium methoxide [88] generated in situ where the authors postulated an associative S_N_2 pathway as they were unable to isolate any bicyclic orthoester intermediate from the crude mixture.

With the aim to get similar neighbouring group protection at C-2 position, it was observed that the neighbouring group participation for the formation of the acetoxonium ion intermediate was more favourable in the locked systems like *o*-substituted benzoyl groups instead of aliphatic protections. Many protecting groups have been developed for the protection of the amine functionality of nucleosides like 2-(benzoyloxymethyl)benzoyl [89] and 2-[(*tert*-butyldiphenylsilyloxy)methyl]benzoyl [90] among others. Sekine and co-workers developed the 2-(azidomethyl)benzoyl (AZMB) group for protecting the hydroxy groups and the exo-amino functionality of the nucleosides [91].

**Scheme 3:**
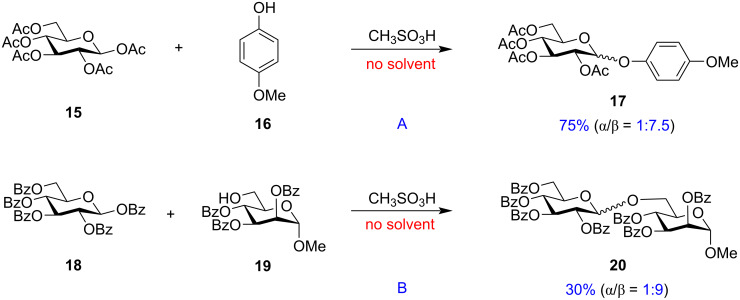
Solvent-free activation [92] of disarmed *per*-acetylated (**15**) and *per*-benzoylated (**18**) glycosyl donors.

Despite their disarming nature, Iadonisi et al. performed glycosylations under solvent-free conditions with poorly reactive disarmed per-*O*-acetylated (**15**) and per-*O*-benzoylated (**18**) glycosyl donors on being activated in air aided by catalytic amounts of a mild promoter, methanesulphonic acid (Scheme 3) [92]. The ester group was capable of conjugating phenol **16** and acceptor **19** to the anomeric position, thereby, making it suitable for glycoconjugate formation. They also reported the glycosylations leading to disaccharide **20** formation in solvent free conditions in significant 1,2-*trans* selectivity. This study holds the potential in improving the various glycosylation reactions making them solvent-free and being more environment friendly without compromising on the stereoselectivity.

Thus, the acetyl and benzoyl group and similar derivatives are the most widely used protecting groups to obtain 1,2-*trans* glycosides in oligosaccharide syntheses. Ester groups in the C-2 position also contribute to the concept of ‘armed–disarmed’ glycosylation wherein C-2 ether-protected ‘armed’ thioglycosides were selectively activated over C-2 ester-protected ‘disarmed’ thioglycosides owing to the electron-withdrawing attribute of the ester carbonyl functionality [93]. However, the concept of ‘armed–disarmed’ protecting groups is often misleading and it has now been accepted that the protecting groups in the far end of the glycoside donor also contribute to the reactivity and stereoselectivity of the produced glycoside, which will be broadly illustrated in the following sections.

**Scheme 4:**
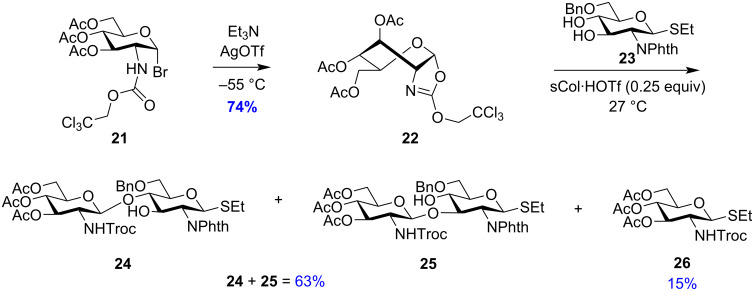
Synthesis of donor 2-(2,2,2-trichloroethoxy)glucopyrano-[2,1-*d*]-2-oxazoline **22** [94] and regioselective synthesis of glycosylation products **24**–**26**.

Similarly, Pertel and co-workers also demonstrated the use of 2-(2,2,2-trichloroethoxy)-2-oxazoline glycosyl donor **22** (Scheme 4) which could be used for stereo- and regioselective glycosylations using extremely mild conditions [94] and requiring low concentrations of the catalyst. In this case the regioselective glycosylation produced products **24**:**25** in a 4:1 ratio in 61% yield. Aglycon transfer was also observed in the reaction producing **26** in 15% yield. The predominance of product **24** also indicates towards a higher nucleophilicity of the C-4 position of the glucoside acceptor **26**.

**Scheme 5:**
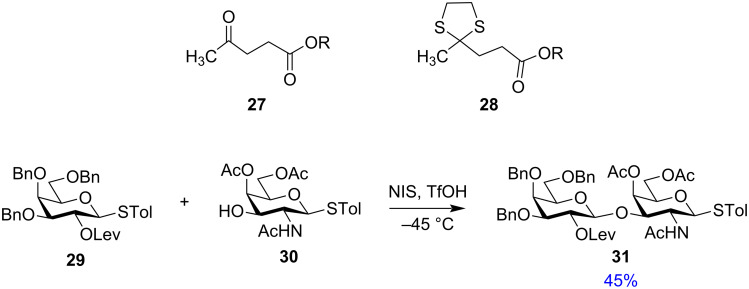
The use of levulinoyl protection for an orthogonal glycosylation reaction.

**Levulinoyl protection:** Levulinic acid (Lev, **27**) as protecting group which can be cleaved using hydrazine hydrate in AcOH, also yields highly selective 1,2-*trans* glycosides [95–96] and was implemented as a substitute for acetyl or benzoyl protection [97–98]. van Boom et al. also showed the use of a masked levulinoyl protecting group, the 4,4-(ethylenedithio)pentanoyl group **28** [99]. Wong and co-workers illustrated the selective activation (Scheme 5) of C-2 levulinoyl-protected thiotolyl glycopyranosyl donor **29** for the synthesis of the disaccharide fragment **31** of fucosyl ganglioside GM1 [100].

**Figure 1:**
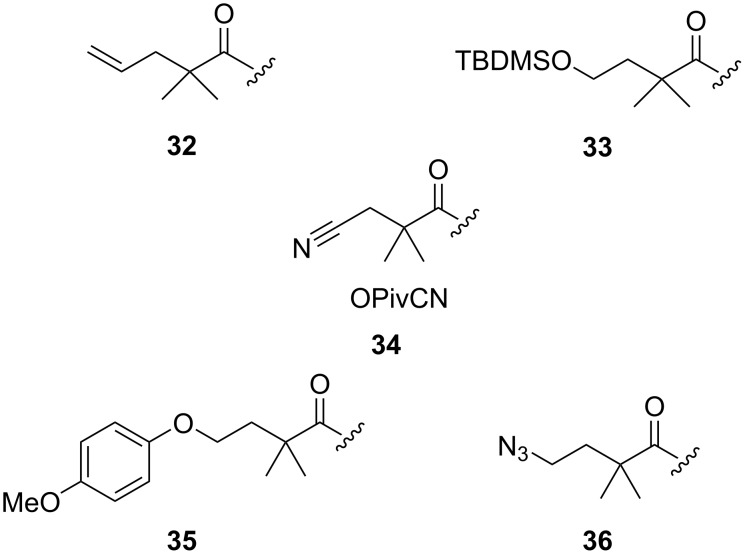
The derivatives **32**–**36** of the pivaloyl group.

**Pivalate protection:** The pivaloyl (OPiv) ester is another such participating ester protection with decreased migratory properties. Moreover, the intrinsic neopentyl property of the ester significantly reduces the probability of nucleophilic attack at the oxocarbenium centre instead of the anomeric carbon, thereby leading to lower amounts of orthoester side products. However, the removal of the pivalate group requires much harsher reaction conditions owing to its steric bulk. Hence, there has been much study to derivatise the pivalolyl ester necessitating milder deprotection conditions. Crimmins et al. in 1998 first introduced the 2,2-dimethylpentenoate protecting group **32** (Figure 1) similar to the pivalate group which showed versatility in its cleavage principle [101]. Hydroboration oxidation of the olefinic bond helped in the removal of the protecting group. On the other hand, dihydroxylation with osmium tetroxide and 4-methylmorpholine *N*-oxide also successfully cleaved the ester reductively by relay-type cleavage. Thus, the tertiary ester could be removed either oxidatively or reductively depending on the sensitivity and requirement of the molecule developed. Similarly, Trost and Hembre devised the 4-(*tert*-butyldimethylsilyloxy)-2,2-dimethylbutanoyl protecting group **33** [102] possessing the steric advantage of the pivaloyl group with the added advantage of it being cleaved with the help of fluoride anions implementing the affinity of the fluoride ion towards the Si atom [103].

**Scheme 6:**
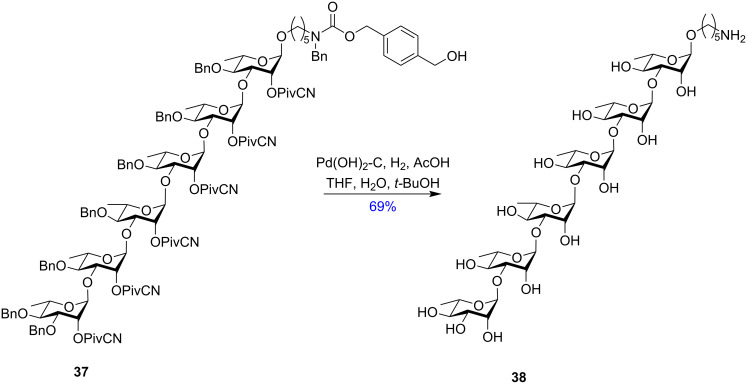
Benzyl and cyanopivalolyl ester-protected hexarhamnoside derivative **37** and its global deprotection protocol [107].

Considering the versatility of the pivaloyl group, Codée et al. in 2013, devised *p*-methoxyphenyl pivalate ester (OMPDMB, **35**) and its azido-functionalised counterpart (OAZDMB, **36**) [104]. These groups also acted as an effective mutually orthogonal protection in oligosaccharide synthesis requiring orthogonal conditions for their respective removal. The *p*-methoxyphenyl group **35** can be removed in mild acidic conditions [105] whereas the azido group **36** is cleaved in basic conditions, using a catalytic amount of KOH for optimum results. Three years later, Codée et al. further derivatised the pivalate ester and introduced the cyanopivaloyl ester protecting group (OPivCN, **34**) as an oligosaccharide protecting group [106]. The cyanopivaloyl group showed high versatility showing the intrinsic advantages of a pivaloyl group while significantly reducing the formation of the orthoester intermediate. It could also be reductively cleaved by hydrogenation in the presence of Pd-C. This deprotection protocol also facilitates an orthogonal deprotection strategy in multistep oligosaccharide syntheses in the presence of benzyl (OBn) groups (Scheme 6). The benzyl and cyanopivaloyl-protected hexarhamnoside derivative **37** was completely deprotected by a single hydrogenation step to yield the product **38** [107]. Compound **37** shows all-1,2-*trans* glycosidic bonds due to the installed pivaloyl group in the C-2 position. This cyanopivaloyl group is also widely used in solid-phase automated oligosaccharide synthesis [107] for the synthesis of oligorhamnoside derivatives. Thus, the pivalate ester protecting group has undergone much changes and derivatisations to optimise its use in oligosaccharide synthesis and to obtain 1,2-*trans* glycosides primarily without the formation of any unwanted orthoester as the by-product. The cyanopivaloyl group has also attracted much interest in machine-assisted oligosaccharide synthesis.

4-Acetoxy-2,2-dimethylbutanoyl (ADMB) esters were reported by Ensley and co-workers [108] having similar properties with the pivalate group. The facile removal of the C-2-ADMB group with a catalytic quantity of diazabicycloundecane (DBU) in methanol at room temperature was the only difference with the pivaloyl group. Protecting the C-2 hydroxy group as ADMB ester yielded 1,2-*trans* glycosides in high yields. However, its participating mechanism is still unclear. So, we reserve our views on placing the use of ADMB as potential neighbouring group participating group. Irrespective of the mechanism, it has been used to prepare pure (1,3)-β-glucan polymers.

**Chloroacetyl and orthogonal protecting groups:** There are other ester-type groups that establish neighbouring group participation when placed in the C-2 position of glycosyl donors, yet differentiate themselves from the other ester-type protecting groups, thereby enabling selective orthogonal deprotection strategies. Such protecting group showing orthogonality with acetyl protection is the chloroacetyl protecting group [109–111]. It renders similar neighbouring group participation by employing the acetoxonium ion, facilitating the 1,2-*trans* glycoside formation in high selectivity. The special feature of this protecting group lies in its orthogonal deprotection in the presence of acetyl or benzoyl protecting groups.

**Scheme 7:**
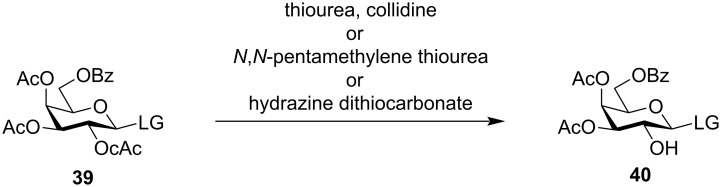
Orthogonal chloroacetyl group deprotection in oligosaccharide synthesis [113].

The chloroacetyl group was found to be particularly labile on the application of thiourea [112], keeping acetyl or benzoyl protection intact (Scheme 7) [113] which contributed to the selective deprotection of sugars in systematic oligosaccharide synthesis. The chloroacetyl group can also be similarly selectively deprotected with other reagents like *N,N*-pentamethylene thiourea [114–115] or hydrazine dithiocarbonate [116–117]. Owing to its versatile selectivity, it has been widely used in oligosaccharide synthesis [118–122] to implement the neighbouring group participation protocol, thereby producing 1,2-*trans* glycosides in high yield and selectivity without affecting any other acetyl or benzoyl groups in the substrate.

**Figure 2:**
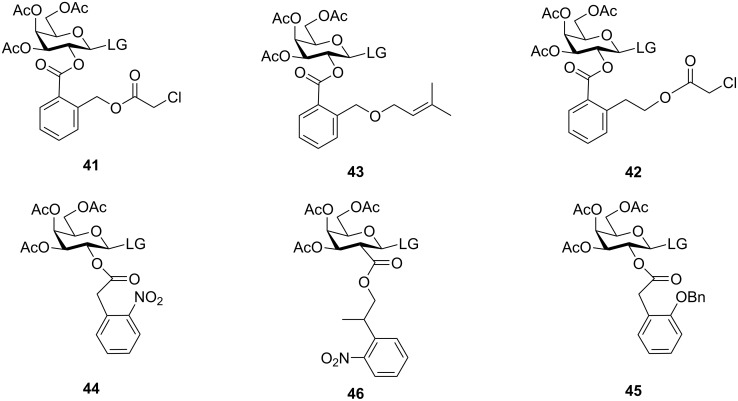
The derivatives of the chloroacetyl group: CAMB protection (**41**) [123], CAEB protection (**42**) [124], POMB protection (**43**) [125], NPAc protection (**44**) [126], BnPAc protection (**45**) [127], and NPPOC protection (**46**) [128].

However, the use of chloroacetyl groups being highly electron-withdrawing in nature, significantly reduces the reactivity of the glycosyl donors, and also undergoes transesterification migration reactions with less nucleophilic acceptors. So, to avoid the undesired migration reactions, Ziegler and Pantkowski developed the 2-(chloroacetoxymethyl)benzoyl (CAMB, **41**) [123] and 2-(chloroacetoxyethyl)benzoyl (CAEB, **42**) [124] groups for the hydroxy-protection in the C-2 position of the glycosyl donors enabling the formation of 1,2-*trans* glycosides with significant yield and selectivity (Figure 2). They could also be selectively cleaved with thiourea without affecting other ester-type protecting groups and implemented the antimigratory attribute of the benzoyl group coupled with the neighbouring group participation property making them a versatile substitute of the chloroacetyl protection for oligosaccharide synthesis.

**Scheme 8:**
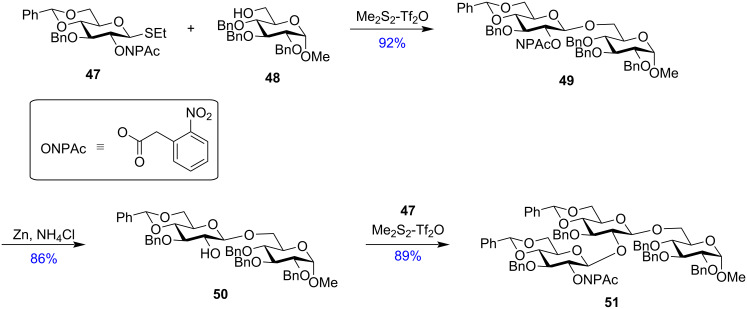
Use of the (2-nitrophenyl)acetyl protecting group [126] as the neighbouring group protecting group at the C-2 position for the formation of 1,2-*trans* glycosides.

However, since the thiourea deprotection is often quite slow in nature requiring the application of heat for a prolonged period, there is always a possibility of extensive side reactions producing undesired side products. Thus, Vatèle devised a temporary protecting group named 2-(prenyloxymethyl)benzoyl (POMB, **43**) which could be selectively deprotected under mild conditions by a combination of DDQ/Yb(OTf)_3_ keeping the other protecting groups, like acetyl, chloroacetyl, benzoyl, pivaloyl intact [125]. Owing to its ester-like properties, this protection group contributed towards the formation of 1,2-*trans* glycosides when installed in the C-2 position. Similarly, the (2-nitrophenyl)acetyl (NPAc, **44**) group has also been used for obtaining 1,2-*trans* glycosides [126]. C-2 NPAc-protected thioethyl donor **47** underwent subsequent glycosylation reactions with different acceptor systems yielding 1,2-*trans* glycosides **49** and **51** in high yields (Scheme 8) depicting the possible formation of the acetoxonium complex as an intermediate.

**Scheme 9:**
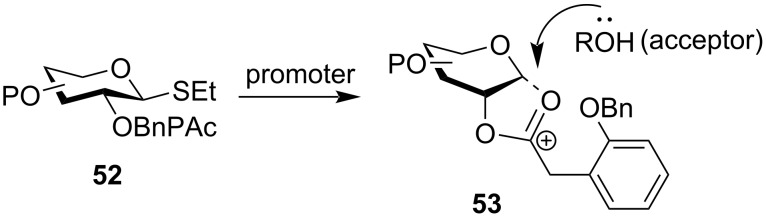
Neighbouring group participation protocol by the BnPAc protecting group [128] in the C-2 position.

Similarly, Mikula et al. also reported a stereodirecting neighbouring protecting group for the C-2 position to obtain 1,2-*trans* glycosides showing orthogonal deprotection according to the need of the compound. They introduced (2-benzyloxyphenyl)acetyl (BnPAc, **43**) as a versatile leaving group showing orthogonality to both acetyl and benzyl protecting groups [127]. This group, when placed in the C-2 position also renders neighbouring group assistance (Scheme 9) with the anomeric carbon thereby forming the acetoxonium intermediate complex **53**. It significantly blocks the α-face of the sugar ring causing the acceptor to attack from the exposed β-face. Moreover, this protecting group is also capable of being cleaved by two possible orthogonal pathways. The first method involves a relay approach by catalytic hydrogenation followed by the application of 1,8-bis(dimethylamino)naphthalene (bDMAN) which selectively cleaves the protecting group while keeping the other ester groups intact. The second procedure for the removal of the BnPAc group is the Zemplén transesterification reaction involving the use of K_2_CO_3_ in MeOH. This method is particularly effective for compounds which are sensitive to catalytic hydrogenation reactions.

In 2012, Calasso et al. demonstrated the use of the CBz group as a stereodirecting protecting group in the C-2 position which helped in the formation of 1,2-*trans* glycosides [129]. Taking their lead, Mikula and co-workers implemented 2-*O*-benzyloxycarbonyl-protected glycosyl donors for the implementation of the stereodirecting property of the carbonate group [130]. The group has shown the formation of 1,2-*trans* glycosides with both carbohydrate-based and non-carbohydrate-based aglycons and carbohydrate acceptors in high yield. Continuing with the carbonyl-mediated glycosylation protocol, Wang et al. showed the application of the photolabile 2-(2-nitrophenyl)propyloxycarbonyl (NPPOC, **46**) group as the 1,2-*trans* stereodirecting group and its use in iterative oligosaccharide synthesis [128].

**Scheme 10:**
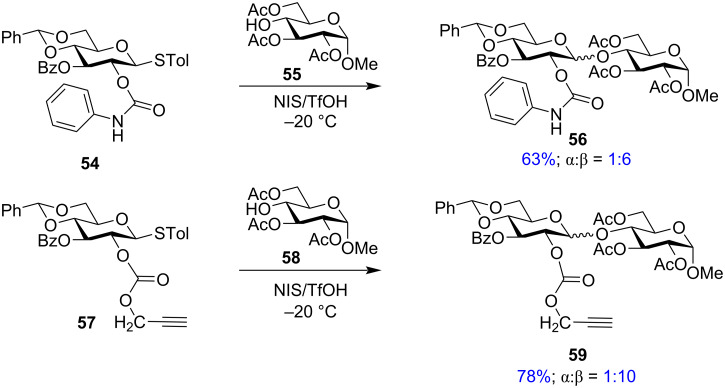
Glycosylation reaction with *O*-PhCar (**54**) and *O*-Poc (**55**) donors showing high β-selectivity [133].

**Other ester-type neighbouring group participation in glycosylation reactions:** Sato et al. worked on the development of new types of ester-protecting groups for the C-2 position of glycosyl donors which can contribute towards 1,2-*trans* glycosylations without affecting the reactivity of the donor. They introduced carbamate ester moieties as alternative protecting groups. The *N*-phenylcarbamoyl (PhCar) moiety showed high stability in the pH range of 1–12 and it could be orthogonally deprotected in the presence of a wide range of other protecting groups like acetyl, benzoyl, acetal, and methoxymethyl [131]. It has been even applied for the first total synthesis of telophiose A [132]. The stereoselectivity and yield obtained in the presence of the PhCar group were improved by using the propargyloxycarbonyl (Poc) protecting group as the C-2 protection in glycoside synthesis [133]. Both the yield and selectivity of the glycosylation reactions were compared with PhCar (**54**) and Poc-protected (**57**) donors with a variety of acceptor glycoside moieties (Scheme 10) which not only showed higher yield (78%) but also a significantly higher anomeric stereoselectivity.

**Scheme 11:**
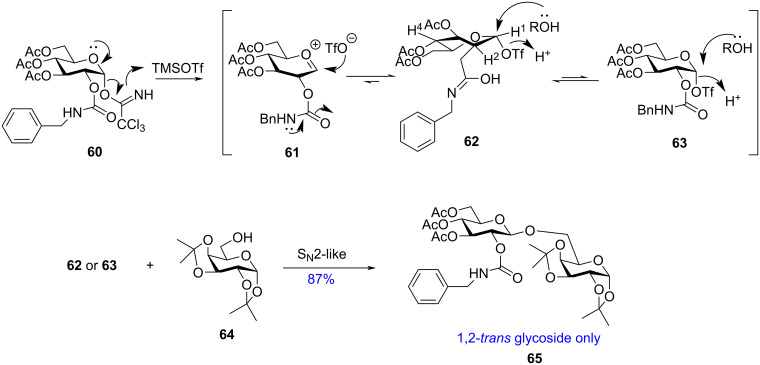
Neighbouring group participation rendered by an *N*-benzylcarbamoyl (BnCar) group [137] at the C-2 position to form 1,2-*trans* glycosides **65**.

With the aim to understand the participating role of the C-2 protecting ‘carbamate moiety’ Liang et al. introduced the *N*-benzylcarbamoyl moiety as the protecting group for the C-2 position [134]. They investigated the role of the C-2 protecting group in Schmidt’s trichloroacetimidate glycosylation with donor **60** which showed excellent yield along with the formation of the 1,2-*trans* glycoside only. They proposed the mechanism with the help of NMR spectroscopy and the studies revealed that the *N*-benzylcarbamoyl (BnCar) group was not responsible for any formation of any oxonium ion in the intermediate steps. Instead, the BnCar group in the C-2 position stabilised the α-triflate (Scheme 11) as proposed by the Deslongchamps model [135–136] which further initiated the formation of 1,2-*trans* or β-glycosides in the subsequent nucleophilic attack by the acceptor [137]. This α-glycosyl triflate was readily converted to its skew-boat conformation **62** which led to the attack of the glycosyl acceptor **64** from the β-face of the sugar pyranoside ring exclusively to form the 1,2-*trans* glycoside **65**. Thus, the *N*-benzylcarbamoyl group helped in the formation of 1,2-*trans* glycosides only without the formation of an acetoxonium ion, although it renders neighbouring group participation to stabilise the 1,2-*cis* triflate intermediate enabling the formation of 1,2-*trans* glycosides. This mechanistic protocol was suitably supported by NMR data showing the formation of the respective intermediates.

**Scheme 12:**
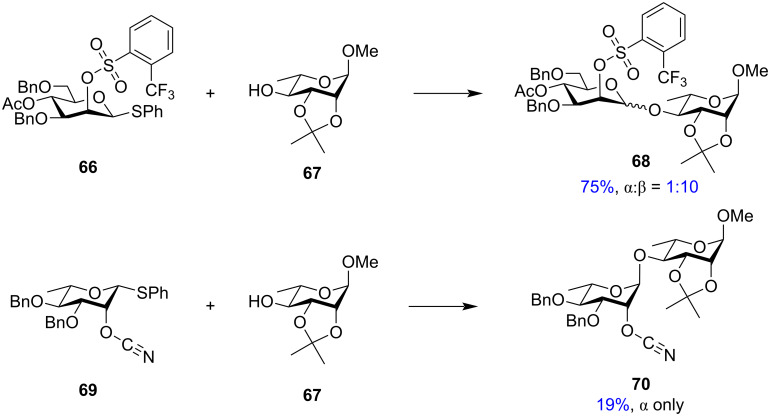
Stereoselectivity obtained from glycosylation [138] with 2-*O*-(*o*-trifluoromethylbenzenesulfonyl)-protected mannosyl donor **66** and 2-*O*-cyano ester protected rhamnosyl donor **69**.

Previously, this stabilisation of a similar α-glycosyl intermediate had been successfully implemented by Crich et al. leading to high β-selectivity in challenging mannose and rhamnose moieties [37]. The moderate yield and β-selectivity with the 2-*O*-sulfonyl-protected mannosyl donor was improved by using a 2-*O*-(*o*-trifluoromethylbenzenesulfonyl)-protected donor which gave a significantly higher yield with greater β-stereoselectivity [138]. The 2-*O*-protected mannosyl unit **66** gave the disaccharide **68** in a 1:10 α:β anomeric ratio (Scheme 12). In addition to the sulphonate groups, Crich et al. also explored the role of 2-*O*-cyanoester as the participating groups at the C-2 position for the formation of β-rhamnoside moieties. However, 2-*O*-cyano ester **69** showed complete α-selectivity when glycosylated with glycosyl acceptor **67** with no isolation of any trace of β-product (Scheme 12).

**Scheme 13:**
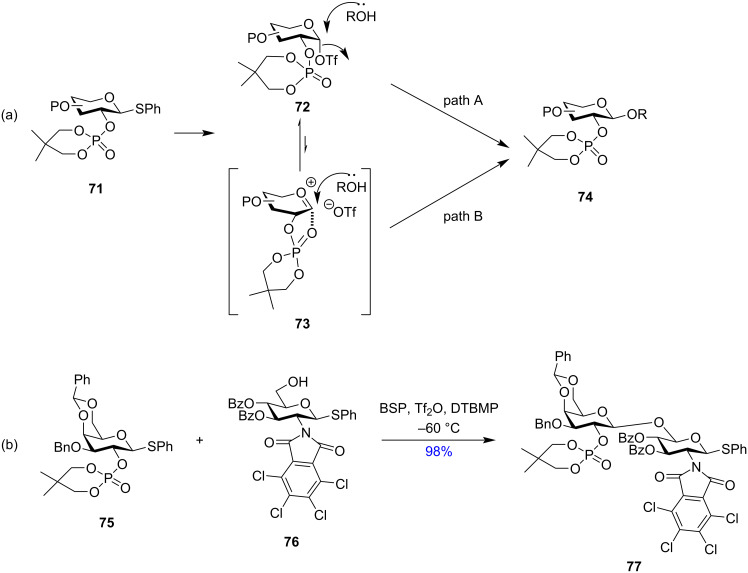
(a) Plausible mechanistic pathway for glycosylation with C-2 DMTM protection [139] and (b) example of a glycosylation showing the stereodirecting property of DMTM.

Based on the same concept and stabilisation of the intermediate α-triflate by the participation of the 2-*O*-protection group in glycosyl donors, Yamago et al. illustrated the use of dialkyl phosphates as the 2-*O*-protecting group (compound **75** in Scheme 13) for the formation of 1,2-*trans* stereodirected glycosyl products **77** when the donor was suitably activated using BSP and Tf_2_O [139]. The role of the 2,2-dimethyltrimethylene (DMTM) phosphate group at the participating C-2 protection was analysed wherein the NMR studies showed no isolation of the corresponding orthophosphate intermediates. However, the NMR analysis showed the formation of covalent α-glycosyl triflates **72** as the intermediate indicating an S_N_2-like mechanistic pathway where the acceptor attacked from the opposite face of the α-triflate intermediates (path A, Scheme 13) which was triggered by the participation of the phosphate group by reverse anomeric effect. However, according to the authors, the phosphate groups acting as a neighbouring participating group forming the corresponding oxocarbenium ion intermediate **73** (path B, Scheme 13) seemed to be more plausible.

#### Non-ester participating protecting groups

Most of the electron-withdrawing ester-type protecting groups are often termed as disarming protecting groups reducing the reactivity of the glycosyl donors. However, on the other hand, these ester groups contribute significantly towards stereodirecting the outcome of the glycosylation reactions. As explained in the previous segments, we see how C-2 protection by ester functionalities leads to the formation of 1,2-*trans* glycosides in high stereoselectivity. So, synthetic glycochemists wanted to design protecting groups which would help in neighbouring group participation and also impart higher reactivity to the glycosyl donors.

**Scheme 14:**
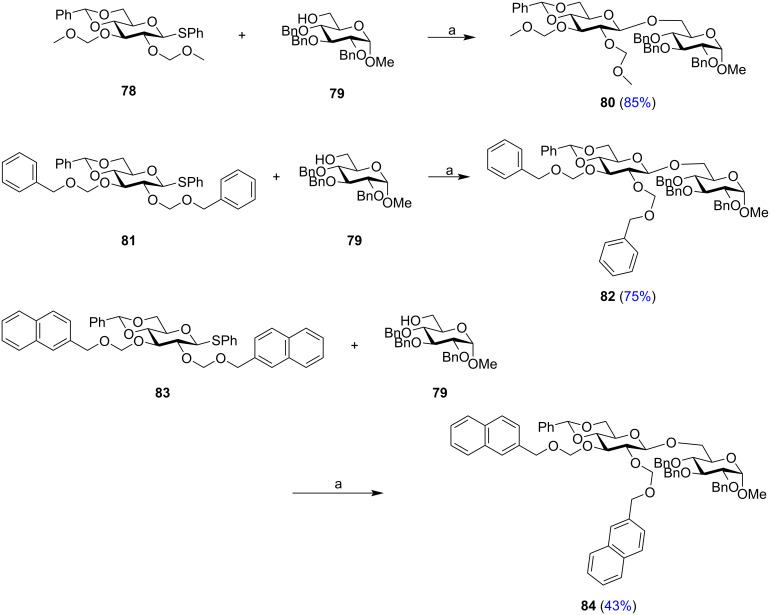
Glycosylation reactions employing MOM **78**, BOM **81**, and NAPOM **83**-protected thioglycoside donors. Reagents and conditions: a) In(OTf)_3_ (1.1 equiv), NIS (1.2 equiv), MS, CH_2_Cl_2_.−78 °C to −30 °C, 0.5 to 1.0 h.

**Alkoxymethyl-type protecting groups:** The alkoxymethyl functionality is a set of new type of protecting groups which when installed in the C-2 position increases the reactivity of the glycosyl donor and also exhibits neighbouring group participation (NGP) to control the 1,2-*trans* selectivity of the glycoside product. Torikai et al. developed a wide range of alkoxymethyl derivatives for directing the stereochemical outcome of the glycosylation reactions. The most significant of the protections include methoxymethyl (MOM) [140], benzyloxymethyl (BOM) and 2-naphthylmethoxymethyl (NAPOM) derivatives [141–142]. Activation of the thiophenyl glycoside donors **78**, protected by the alkoxymethyl groups at the C-2 position by a combination of NIS and In(OTf)_3_, followed by the nucleophilic attack of the acceptor glycoside **79** produced 1,2-*trans* glycosides (Scheme 14). The 2-O-MOM **78** and 2-O-BOM **81**-protected glycosides afforded 1,2-*trans* glycosides **80** and **82** exclusively in 85% and 75% yield, respectively. On the other hand, Torikai’s group also implemented the NAPOM protecting group **83** introduced in the C-2 position which furnished the required 1,2-*trans* glycoside in moderate 43% yield.

**Scheme 15:**
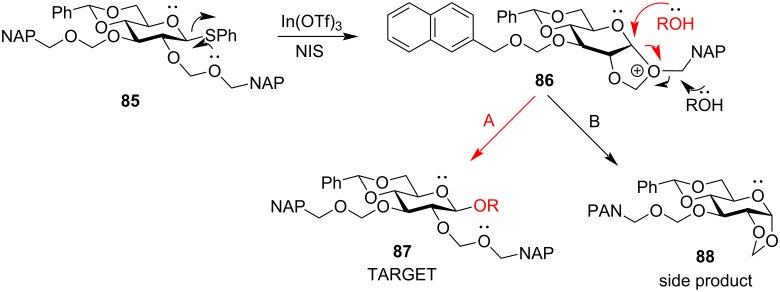
Plausible mechanistic pathway for alkoxymethyl-protected glycosyl donors. Path A. Expected product and path B, isolated side product.

However, a five-membered cyclic acetal **88** as the side product was obtained which led to the determination of the plausible mechanism involving participation of the alkoxymethyl group of the C-2 position (Scheme 15). Path A in Scheme 15 designates the expected participating route for 1,2-*trans* glycoside synthesis. Path B designates the route for the formation of the cyclic ester as the side product which is only possible if the mechanism proceeds through the formation of an oxocarbenium intermediate **86**. This has been effectively shown by Kulkarni and co-workers who used a 9-anthracenylmethyl group in the C-2 position which rendered neighbouring group participation effectively via a favourable π–π interaction between the p-orbitals of the stabilised oxonium ion intermediate and the aromatic ring of the protecting group to yield 1,2-*trans* selective products. Increasing the aryl ring size was also an additive by exhibiting steric hindrance towards the attack of the incoming nucleophile [143].

Tanaka et al. also showed the use of the phthalimidoxy functional group at the C-2 position leading to the 1,2-*trans* glycosides via the formation of six-membered carbenium ion intermediates [144]. However, C-2 phthalimidoxy-protected trichloroacetimidate glycosyl donors gave higher 1,2-*trans* selectivity in comparison to C-2 phthalimidoxy-protected *N*-phenyl 2,2,2-trifluoroacetimidate glycosyl donors.

**Scheme 16:**
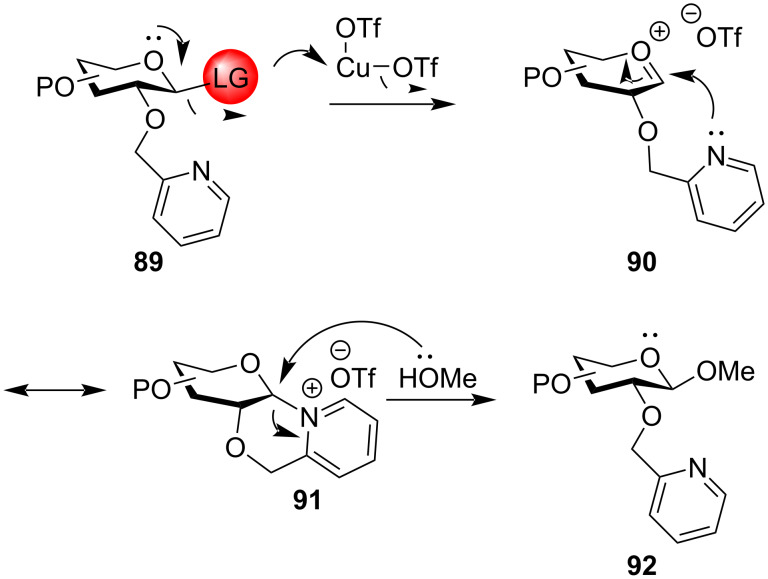
Plausible mechanistic pathway for alkoxymethyl-protected glycosyl donors [147].

**Ether-type participating protecting groups:** In the absence of neighbouring acyl-type protecting groups in the C-2 position in glycosyl donors, on the elimination of the activated leaving group from the anomeric position, the flattened oxocarbenium ion formed causes the incoming nucleophilic acceptor to attack from either the β or the α-face of the sugar ring, thereby leading to the formation of a mixture of 1,2-*cis* and 1,2-*trans* glycosides. In this respect ether-type non-participating protecting groups like benzyl (OBn), *p*-methoxybenzyl (OMBn), and allyl (OAll) are implemented as the temporary protection in the C-2 position in order to obtain 1,2-*cis* glycoside products. Moreover, the ether-type groups are less electron-withdrawing than the ester groups [55,145–146] making the corresponding glycosyl donors more reactive (armed) than the corresponding donors with ester group protection. However, the use of ether groups works on the protocol of elimination 1,2-*trans* selectivity instead of actual stereodirecting the glycosylated product to 1,2-*cis* product. There have been reports of a wide range of ether-type protecting groups which exhibit neighbouring group participation in order to obtain 1,2-*trans* glycosylated products exclusively. One such widely implemented protecting group is the 2-*O*-picoloyl or 2-pyridylmethyl (Pic) protecting group. Demchenko and co-workers utilised the arming participating property of this Pic group for C-2 protection in thioglycoside donors in orthogonal glycosylation reactions [147]. For the mechanism involved (Scheme 16), after the promoter-assisted departure of the leaving group, the lone pair of electrons of the nitrogen atom of the pyridine fragment of the Pic group participates to form a stable oxocarbenium intermediate **91** through the formation of a six-membered ring. The glycosylation mechanism proceeds via an S_N_2-like pathway through the formation of tightly bound ion pair, where the incoming nucleophilic acceptor molecule attacks from the β-face of the pyranoside ring to form the 1,2-*trans* glycosides **92** exclusively. Thus, Demchenko et al. elaborated the use of such armed participating neighbouring groups for the consecutive synthesis of 1,2-*trans* glycosidic bonds. This protocol was also successfully implemented in orthogonal glycosylations with the disarmed glycosides and the same anomeric leaving group enabling further tuning of the protecting group manipulations in oligosaccharide synthesis.

**Scheme 17:**
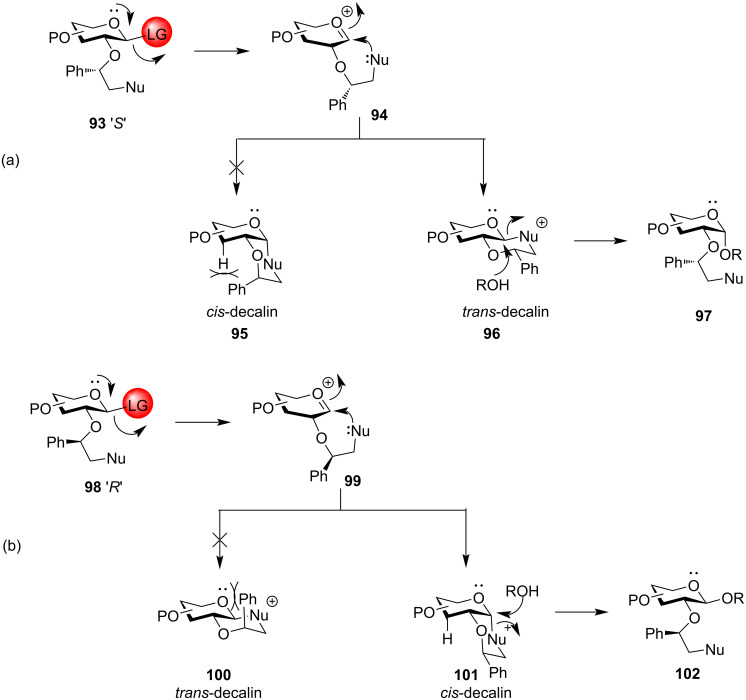
A. Formation of α-glycosides and B formation of β-glycosides by using chiral auxiliary neighbouring group participation [149].

Another versatile participating ether-type chiral auxiliary group for protection of the hydroxy group in the C-2 position was devised by Boons et al. in 2005 which opened a new avenue in oligosaccharide synthesis. Auxiliary group indicates a substituted ethyl protection which has a nucleophilic centre that can donate electrons in the tartgeted reaction [148]. A proper modulation of the stereochemistry of the chiral auxiliary group allows to obtain both the 1,2-*cis* and 1,2-*trans* stereoselective glycoside product (Scheme 17) [149]. An *O*-2 chiral auxiliary group in the glycoside donor interacts with the anomeric carbon to produce a decalin intermediate by neighbouring group protection. Boons et al. demonstrated that the auxiliary group with *S* stereochemistry **93** leads to the formation of *trans*-decalin **96** only due to the instability of the alternate *cis*-decalin **95** owing to the unfavourable steric hindrances exhibited by the axial phenyl substituent. Nucleophilic attack of the acceptor, ROH on this *trans*-decalin system **96** yields 1,2-*cis* glycosidic product **97**. On the other hand, when using an auxiliary group with *R* stereochemistry **98**, the *trans*-decalin system **100** experiences unfavourable steric hindrances thereby helping in the formation of 1,2-*cis* decalin **101** facilitating the formation of 1,2-*trans* glycosidic product **102**. This protocol was further demonstrated successfully by the same research group by using the easily available *R* and *S* enantiomers of the first-generation chiral auxiliary, ethyl mandelate. Similarly, a (1*S*)-phenyl-2-(phenylsulfanyl)ethyl moiety in the C-2 position led to the stereoselective formation of 1,2-*cis* glucoside and 1,2-*cis* galactoside in high yield by the formation of a quasi-stable anomeric sulphonium ion intermediate [150]. In continuation with the use of auxiliary protecting groups as the O-2 protection for their stereodirecting effect, Boltje et al. demonstrated a series of six ether, tertiary amide, and phosphine oxide-based auxiliary O-2 protecting groups [151]. The results indicated increased 1,2-*cis* selectivity with tertiary amide and phosphine-based protecting groups in comparison to an ether-based group.

Thus, it was observed that the 1,2-*cis* selectivity obtained in the glycosylation reactions increased with the increase of electron density on the sulphonium ion and corresponding decrease of electron density on the oxocarbenium ion. This was illustrated by Boltje et al. demonstrating 1,2-*cis* stereoselective glycosylation reactions with 2,4,6-trimethoxythiophenyl protection and 2,4-dichlorobenzyl-protection in the O-2 position [152]. In sync with the protocol of chiral stereodirecting protecting groups by Boons, Fairbanks and co-workers also implemented the trimethoxy-substituted thiophenonium group as a protecting group [153] for the C-2 position which successfully formed a six-membered ring intermediate via neighbouring group participation to form completely selective 1,2-*cis* glycosylated products with a range of carbohydrate acceptors. The formation of ring intermediate and glycosylating species was confirmed by low temperature NMR spectroscopy.

**Scheme 18:**
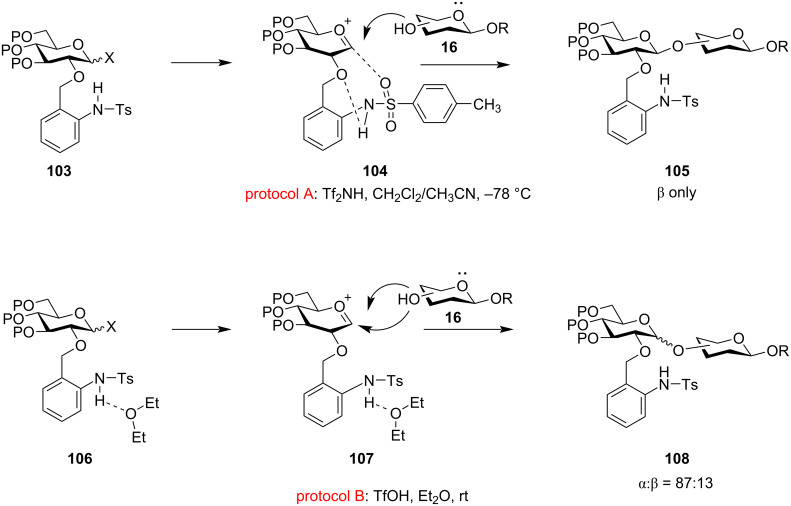
Bimodal participation of 2-*O*-(*o*-tosylamido)benzyl (TAB) protecting group to form both α and β-isomers [154–155].

Similarly, Ito et al. also introduced the concept of using 2-*O*-(*o*-tosylamido)benzyl (TAB)-modified donor **103** for the stereocontrolled synthesis of both 1,2-*cis* and 1,2-*trans* glycosides under modified reaction conditions [154–155]. They demonstrated that the use of triflimide (Tf_2_NH) in solvents like CH_2_Cl_2_, acetonitrile or toluene at −78 °C yielded exclusively the 1,2-*trans* stereoselective glycoside product **105** (protocol A, Scheme 18), while the use of triflic acid (TfOH) in ether as the solvent at ambient temperature conditions (protocol B, Scheme 18) gave product **108** with higher 1,2-*cis* selectivity. In protocol A, the tosylamide forms an intramolecular hydrogen bonding with the benzylic oxygen forming a quasi-bicyclic intermediate acting as a 1,2-*trans* directing protecting group. Thus, following subsequent formation of the oxocarbenium ion **104**, neighbouring group participation is exhibited by the sulphonamide oxygen thereby giving complete 1,2-*trans* stereoselectivity by facilitating the attack of the acceptor molecule from the non-hindered β-face of the sugar ring. However, in protocol B, the use of participating solvents like ether disturbs the intramolecular hydrogen bonding, as in **107** thereby generating a non-participating form as an intermediate. Thus, the acceptor easily attacks from the α-face of the sugar ring, and thereby, leading to the product with higher 1,2-*cis* stereoselectivity (87:13). A similar bimodal protocol was also implemented for the formation of α and β-mannosides using an *O*-2 TAB-protected glycosyl donor providing moderate to high yields [156].

**Scheme 19:**
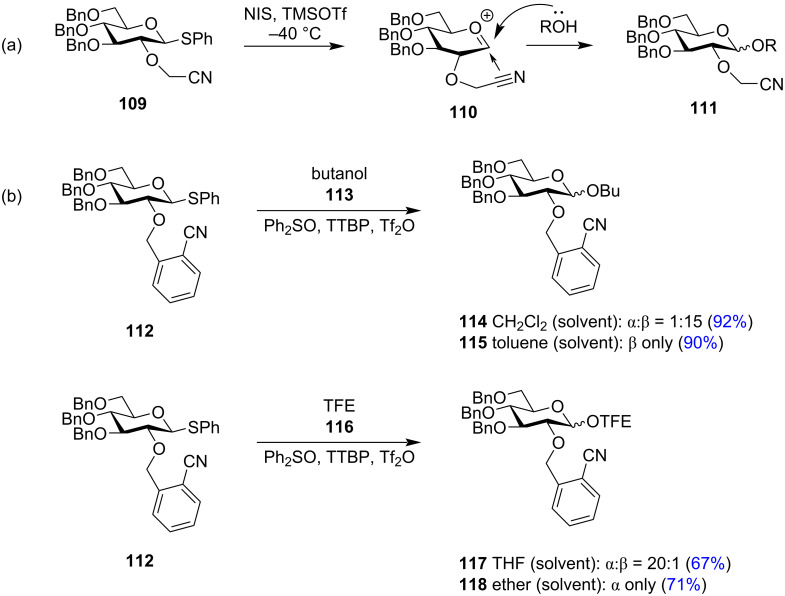
(a) 1,2-*trans*-Directing nature using C-2 cyanomethyl protection and (b) the effect of acceptors and solvent on the same C-2 cyanobenzyl-protected glycoside donor [159].

With the aim to derive more ether-type ‘arming protecting groups’ for 1,2-*trans* stereodirecting property, the C-2 cyanomethyl ether group was implemented as a potential participating group for glycosylation reactions (Scheme 19a) [157–158]. The oxocarbenium ion **110** formed in the process of the glycosylation reaction is stabilised from the α-face by the nitrile on the methyl ether via its π-electrons. This enables the attack of the approaching acceptor molecule from the β-face thus facilitating the formation of 1,2-*trans* glycosylated product **111**. However, similar studies done with the cyanobenzyl group demonstrated the role of the acceptor molecule in determining the stereoselectivity of the produced glycoside molecules (Scheme 19b) [159]. The S_N_2 glycosylation of the nitrilium ion-type intermediate with the acceptor molecule yielded the 1,2-*trans* product while H-bonded glycosylation of the acceptor molecule gave the 1,2-*cis* exclusive product. Corresponding to this protocol, butanol (**113**) as acceptor with C-2 cyanobenzyl-protected glycoside donor **112** gave solely the 1,2-*trans* product **114** or **115**, while TFE (2,2,2-trifluoroethanol, **116**) as acceptor gave the 1,2-*cis* product **117** or **118** primarily. The exclusive formation of the 1,2-*trans* and 1,2-*cis* product was achieved with butanol and TFE, respectively, by correspondingly changing the solvent system to toluene and ether, respectively. Thakur and co-worker implemented the use of a nitrogen-rich tetrazole-linked ether group for a similar 1,2-*trans* stereodirecting property [160]. In this respect, they introduced the use of (1*N*/2*N*)-methylated tetrazole methyl (MeTetMe) ethers as a C-2 protecting group for glycosyl donors which was used for glycosylation reactions in combination with the PIFA-TfOH reaction system. MeTetMe was particularly effective and could be orthogonally removed under Birch reduction conditions.

#### Inference on neighbouring group participation

Thus, we see that the electron-withdrawing groups like an ester functionality in the C-2 position contributed significantly in obtaining 1,2-*trans* glycosides by virtue of the formation of dioxolenium ions by neighbouring group participation. This method is most widely implemented by glycochemists due to its productivity, reproducibility, regio- and stereoselectivity, and yield. However, ester groups also exhibit certain limitations corresponding to their use in synthetic carbohydrate chemistry. These electron-withdrawing groups reduce the inherent reactivity, and thereby, disarming the participating glycosyl donors. Formation of an orthoester side-product also reduces the yield of the reaction in certain cases. They also exhibit significant migration properties leading to the formation of a mixture of glycosylated products [161]. As a solution to this, there have been numerous studies to use ‘arming’ ether protecting groups in the C-2 position with neighbouring group properties to obtain 1,2-*trans* glycosides without compromising the reactivity of the glycosyl donors. The use of chiral and achiral auxiliary groups also participates through various pathways including solvent participation and H-bonding assisted glycosylation protocols enabling the modulation of the stereoselectivity of the glycosylation reactions according to the requirement to obtain either 1,2-*cis* or 1,2-*trans* glycosides. It is, however, to be kept in mind that although protecting groups in the C-2 position contribute significantly in obtaining the required stereochemistry of the glycosylated product, other factors like nature of the acceptor, temperature, activation conditions, and solvent also are important factors that should be considered.

### Remote group participation

So far, we explained the role of participating groups present at the C-2 position which is vicinal to the anomeric carbon atom to obtain the required stereocontrolled product. The presence of participating groups in the distal C-3, C-4, or C-6 position other than the C-2 position affecting the stereochemistry of the produced glycosidic bond is more commonly termed as ‘remote participation’ of protecting groups on glycosylation. However, to categorise the role of the distant participating groups, it is essential that there is a non-participating group in the C-2 position exhibiting no effect on the stereochemistry of the produced glycosidic bond. The concept of ‘remote participation,’ however, is highly debated and a much studied topic in synthetic glycochemistry [162–164]. The effects attributed by the remote groups may also be the summative effect of other factors like steric hindrance, conformational hindrance, or other stereoelectronic factors. However, in this article we have tried to show all the possible effects exhibited by the protecting groups in the C-3, C-4 and C-6 position of the glycopyranoside rings which may contribute to stereodirecting the outcome of the glycosylation reactions with an intention to give an overview of the various theories that may be applicable in synthesizing the required stereoselective glycosidic bond. The readers are advised to verify the results with an inquisitive and analytical knowledge to conclude the effects of remote participation on glycosylation reactions.

The anomeric reactivity of the glycosyl donors has also been reported to be influenced by the conformation of the protecting groups or side chains in the remote C-3, C-4, and C-6 positions. The presence of ester-type protections at C-6 having electron-withdrawing ‘disarming’ properties exhibits an increased concentration of the less reactive ‘*trans, gauche* (*tg*)’ conformation compared to the ether protections, thereby reinforcing the electron-withdrawing properties of the ester functionalities which reduces the reactivity of the donors [165]. However, a change of the protecting side group from ether to ester in the remote C-4 position of galactopyranosides, causes a slight reduction of the proportion of the less reactive *tg* conformation [166]. Thus, it neutralises the disarming properties of the ester side chains causing slight increase in the anomeric reactivity. This contributes towards the activation of the thioglycoside donors or S_N_2-type displacement reaction of the glycosyl halides. It was concluded by Crich and Whitfield that the ring conformation of the produced intermediates in the glycosylation reaction are largely responsible for the reactivity and the stereochemistry of the produced glycoside products [167–168] which will be explained in the following sub-sections.

#### Ester-type remote protecting groups

**Scheme 20:**
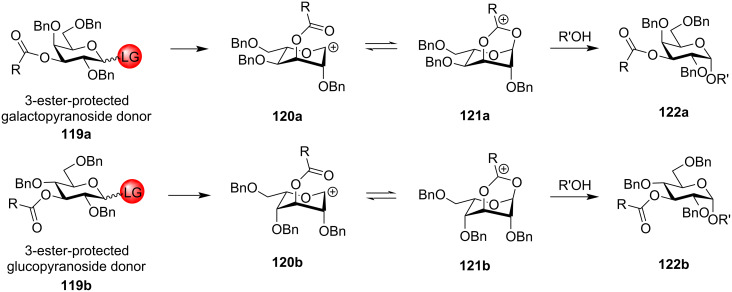
1,3-Remote assistance by C-3-ester protection for gluco- and galactopyranosides to form 1,2-*cis* glycosidic products [172].

**Scheme 21:**
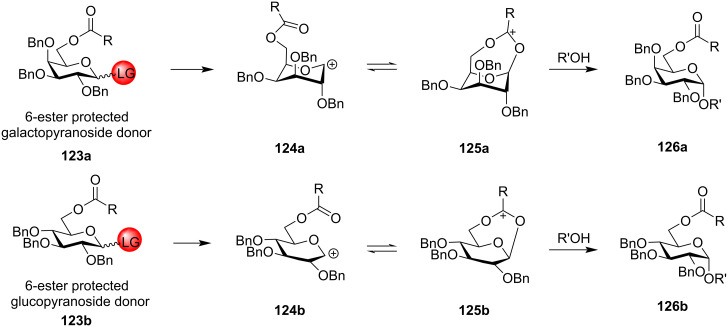
1,6-Remote assistance by C-6-ester protection for gluco- and galactopyranosides to form 1,2-*cis* glycosidic products.

Boons et al. first delineated the possible remote participation of the distal ester protection in the C-4 position of galactopyranoside donors resulting in the α-selectivity which was further reinforced by changing the solvent system to 1,4-dioxane/toluene [169]. Previously, highly selective 1,2-*cis* fucosylations by remote participation of the distal O-4 position had also been reported [170–171]. The presence of ester-type protecting groups in the distal position also affected the stereochemistry of the glycosidic product, in a similar way like C-2 neighbouring group participation with similar vicinal protection. However, the C-2 neighbouring group assistance by an ester-type group requires the formation of the fused bicyclic acetoxonium intermediate **11** (Scheme 2). However, in the case of the presence of an ester-type protecting group in the distal C-3, C-4 and C-6 position, a bridged bicycle is formed as the intermediate by virtue of their remote group assistance [172]. As a general mechanistic explanation, according to Scheme 20 and Scheme 21, a participating acyl group at the C-3 position and C-6 position of both gluco- and galactopyranosides forms the bicyclic intermediates **121a**/**b** and **125a**/**b**, whereby the sugar ring undergoes a certain conformational distortion. Thus, the remote acyl groups are able to interact with the anomeric carbon, thereby facilitating the attack of the acceptor molecule in a specific stereodirected manner enabling the formation of 1,2-*cis* glycosides **122a**/**b** and **126a**/**b**.

**Scheme 22:**
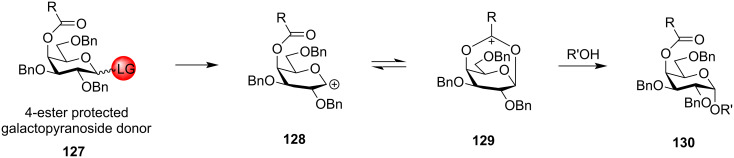
1,4-Remote assistance by C-4-ester protection for galactopyranosides to form 1,2-*cis* glycosidic products.

However, when the acyl group is in the C-4 position, the bicyclic intermediate is produced only in the case of galactopyranosides (Scheme 22). In the case of galactose donor **127**, an acyl protecting group in the C-4 position produces the intermediate **129** which leads to the S_N_2-type reaction forming 1,2-*cis* glycoside **130** as the major product. Participation of the remote C-4 position on the glycosylations was first experimentally proved by Yu et al. through the formation of a bridged bicyclic intermediate [173]. This observation led to the conclusion that the conformation of the oxocarbenium ion intermediate is largely responsible for the stereochemistry of the obtained glycosidic output [174]. Thus, remote group participation provides an opportunity to utilise the relative stereochemistry of the protecting groups to control the facial selectivity in glycosylation reactions [119,175–180].

However, electron-withdrawing, potentially participating protecting groups at O-3, O-4, and O-6 positions make it difficult to distinguish their electron-withdrawing effects from their remote participatory effect on the outcome of the stereochemistry of the product. Specifically, in the case of mannosides, general concepts indicate that the remote participation would facilitate the formation of 1,2-*trans* or α-mannosylation, while an electron-withdrawing effect would facilitate 1,2-*cis* or β-mannosylation. While the group of van Boeckel et al. opposed the concept of remote participation by 3-O and 4-O acetyl groups [181], Boons and co-workers reported that an electron-withdrawing nature of ester groups in the remote group, coupled with the use of solvents like 1,2-dioxane/toluene facilitated the formation of 1,2-*trans* glycosides by intermediate stabilisation of the oxocarbenium ion [169]. This was further reinstated by Nifantiev et al. who inferred that the electron density on *p-*substituted benzoyl groups highly induced 1,2-*trans* selectivity in galacto- and fucosylation reactions [182]. However, no evidence was found which showed contradiction of the glycosylation results in the absence of remote participation. Thus, there have been various reports both in favour and against the potential remote participation of electron-withdrawing protecting groups in the O-3, O-4, and O-6 remote positions.

**Scheme 23:**
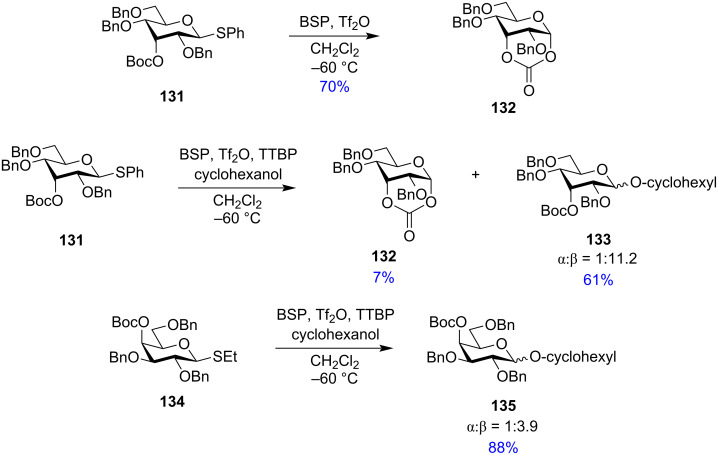
Different products obtained on activation of axial 3-O and equatorial 3-O ester protected glycosides [188].

There has been a wide array of studies to detect the dioxolenium ion formed by the remote ester protecting groups using cryogenic vibrational spectroscopy [183] and solution phase NMR spectroscopy [184–185]. Infrared ion spectroscopy (IRIS) [186], density functional theory (DFT) calculations, and model glycosylation experiments [187] showed that the remote group participation effect was highly dependent on the position of the protecting groups. However, for possible interactions of the remote groups, the essential change of the ring conformation from ^4^*C*_1_ to ^1^*C*_4_ structure has been exhibited by the interaction of an equatorial participating group in the C-3 position with the anomeric carbon [157–158]. Efforts to trap the oxocarbenium ion intermediate have been made by using the di-*tert*-butoxycarbonyl (Boc) group in the C-3 and C-4 positions. To verify whether participation by distal ester groups actually effects the stereochemistry of the produced glycosidic bond, Crich et al. showed the influence of Boc protection in each of the O-3, O-4, and O-6 position (Scheme 23) [188]. The *axial* 3-O ester **131** on activation by BSP, Tf_2_O at −60 °C led to the sole isolation of the cyclic carbonate **132** in 70% yield in the absence of any attacking nucleophile. However, upon the addition of the mild, non-nucleophilic base tri-*tert*-butylpyrimidine (TTBP) and an external acceptor cyclohexanol led to the formation of the glycoside **133** in 61% yield with an α/β anomeric ratio being 1:11.2 along with the isolation of cyclic carbonate ester **132** in 7% yield. However, the equatorial 3-O ester sulphonate on activation by BSP, Tf_2_O and TTBP in the presence of the attacking nucleophile exclusively gave the α-anomer with no isolation of any cyclic carbonate spanning positions 1 and 3 of the pyranose ring. Thus, no evidence could be found for any participating effect of remote ester protection in equatorial position and thus Crich et al. devised that the formation of the α-glycoside was not due to any participatory effect of the equatorial O-3 ester protection. Similarly, also no evidence was found for the participatory effect for axial or equatorial 4-O ester and 6-O ester protection. Activation of axial 4-O Boc-protected glycoside **134** in the presence of a nucleophile gave the glycoside product **135** as an anomeric α:β mixture of 1:3.9 with slight excess of β-isomer. This observation contradicted the possible distal group participation theory. All these findings led Crich and co-workers to conclude that stereoselectivity may be the summation of many other effects attributing their stereodirecting impact on the produced glycosides.

On the other hand, there have also been certain reports demonstrating the formation of the bridged bicyclic orthoester-type products owing to the interaction of remote participating groups with the anomeric carbon [173,189]. It has been argued that the inability to detect the intermediate glycosyl cations by NMR spectroscopy was because the lifetime of the glycosyl cations is usually shorter than the relaxation time of NMR spectroscopy [184]. Boltje et al. could characterise the low populated bicyclic reaction intermediates by the help of chemical exchange saturation transfer (CEST)-NMR. In a recent report, the group also characterised elusive rhamnosyl dioxanium ions, validating a significant C-3 acyl participation in the gas-phase using IRIS and in the solution-phase using CEST NMR [190]. Thus, the concept of remote participation continues to remain a highly discussed and controversial topic for carbohydrate synthetic chemists with groups giving evidences pertaining to both for and against the concept.

**Scheme 24:**
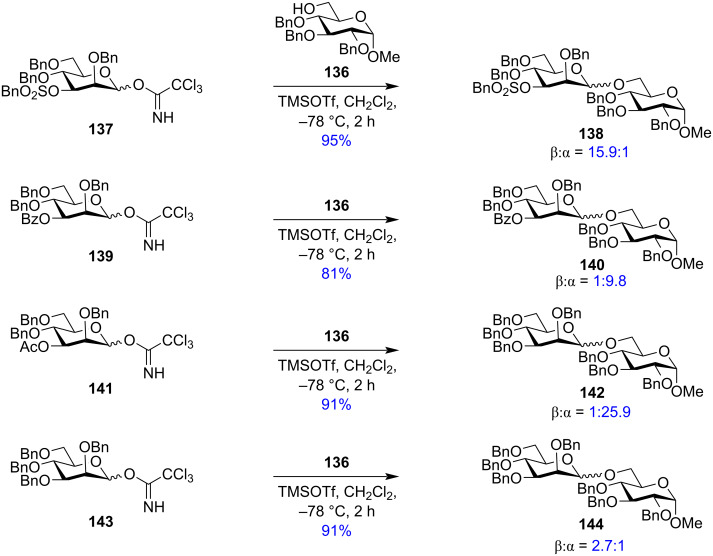
The role of 3-*O*-protection on the stereochemistry of the produced glycoside [191].

However, to delineate the role of remote protecting groups in mannose sugar units with axial C-2 position, in another report in 2009, Kim et. al reported the role of remote O-3,O-6 electron-withdrawing acetyl groups in stereodirecting the product of the mannosylation reactions [191]. They reported that non-participating strongly electron-withdrawing groups in the C-3 position like benzylsulphonyl-protected glycoside **137** on activation by TMSOTf in the presence of benzyl-protected glycoside **136** as the acceptor, triggered the formation of the product **138** with large excess of 1,2-*cis* or β-mannosides (Scheme 24). But the replacement of the C-3 protection with less electron-withdrawing participating acyl groups like benzoyl (**139**) and acetyl (**141**) led to the formation of glycosides **140** and **142**, respectively with the α anomer predominating. The most α-directing nature was observed with an O-3 acetyl group which produced the resulting glycoside **142** as α-anomer predominantly. This participatory nature was distinctly absent when the O-3 acyl group was replaced by a benzyl group where an anomeric mixture of glycosides was obtained. Similar β-directing effects were observed with strong electron-withdrawing protecting groups in the distant C-6 position while the 6-O acetyl group exhibited a strong participatory nature to yield almost exclusive the α-anomer.

**Scheme 25:**
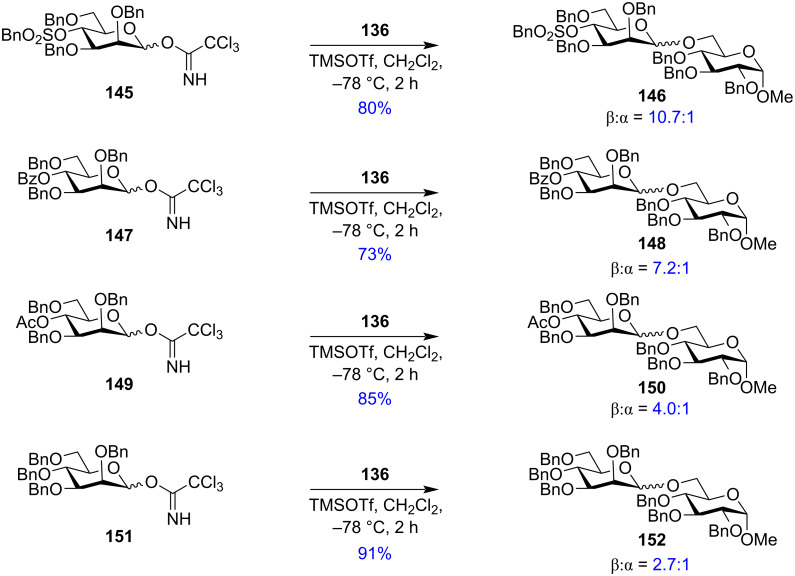
The role of 4-*O*-protection on the stereochemistry of the produced glycosides.

However, a slight difference in participatory effect was observed for O-4 protection (Scheme 25). The group reported that strongly electron-withdrawing, non-participating groups at the O-4 position like benzylsulphonyl groups in **145** rendered strongly β-selective mannosylation reactions with a β:α ratio of 10.7:1 for **146**, respectively, while potentially participating weakly electron-withdrawing groups like *p*-nitrobenzoyl, benzoyl (**147**), and acetyl (**149**) groups also favoured β-selectivity to form products **148** and **150** but with decreased selectivity. However, glycosyl donor **151** without any electron-withdrawing group in the C-4 position gave a mixture of α/β products. Thus, although the electron-withdrawing protecting groups in the C-4 position rendered some effect on the anomeric selectivity, the α-directing effect of the O-4 acetyl protection was not as dominant as in the O-3 and O-6 position. Thus, it was postulated that remote electron-withdrawing groups in the O-3, O-4, and O-6 position showed β-directing nature while an acetyl group in the O-3 and O-6 position showed strong α-selectivity due to remote participation.

**Scheme 26:**
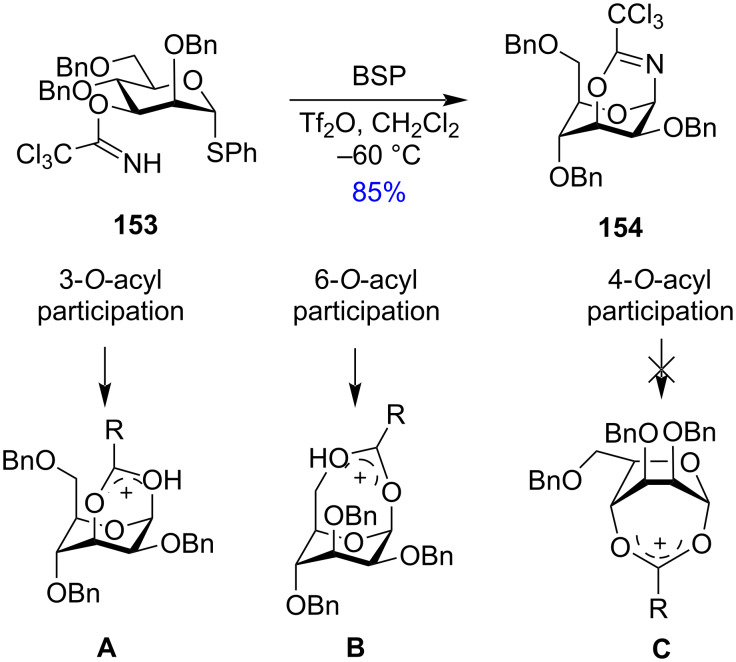
Formation and subsequent stability of the bicyclic oxocarbenium intermediate formed due to remote participation of acetyl groups.

As shown previously by Crich and co-workers [188], although there was no isolation of cyclic products in the trapping experiments with 3-*O*-Boc protection, a stable bicyclic product was obtained having a six-membered trichlorooxazine ring which provided possible evidence of the remote participatory effects of the C-3 acyl protection (Scheme 26). ^1^H NMR showed that the O-3 trichloroacetimidate mannopyranosyl donor **153** in ^4^*C*_1_ conformation underwent the change of ring conformation to ^1^*C*_4_ to interact with the anomeric carbon by forming the bridged bicyclic product **154** (Scheme 26).

Comparison of the relative stabilities of the cyclic intermediate produced due to remote participation by 3-*O*, 4-*O*, and 6-*O*-acyl groups showed that the 3-*O*-acyl groups generated a relatively stable bicyclic six-membered ring dioxocarbenium ion intermediate (**A**, Scheme 26) leading to a maximum remote participatory effect by the 3-*O*-acyl groups, whereas 6-*O*-acyl groups generated a less stable seven-membered dioxocarbenium ion (**B**, Scheme 26). Participation of the 4-*O*-acyl group gave the least stable seven-membered dioxocarbenium ion (**C**, Scheme 26). While 3-*O* and 6-*O*-acyl participation formed a ^1^*C*_4_ ring conformation, the 4-*O*-acyl participation converted the ring conformation to boat-type (**C**, Scheme 26). This proved that mannosylation reactions with 4-O-acyl groups did not involve the intermediate **C**. All these studies and observations provided solidarity to the concept of remote participation by the acetyl groups of the distal O-3 and O-6 positions wherein Kim et al. [192] gave evidence in favour of the concept of distal participation of the protecting groups in glycosylation reactions.

It has also been observed that addition of an extra remote acyl group in the O-3 position in 4,6-di-*O*-acetylglucopyranosides gave higher α-stereoselectivity in comparison with mono-6-*O*-acetyl-protected donors [192]. This phenomenon helped to confirm and ascertain the importance of remote group participation in streamlined oligosaccharide synthesis [193–194]. Remote participation by participating groups at the 3-position has also been demonstrated in 3-*O*-acetylated 4,6-*O*-benzylidene-protected mannopyranosyl donors which exhibited significant decrease in its β-stereoselectivity and increase in the yield of α-glycoside products [193,195].

**Scheme 27:**
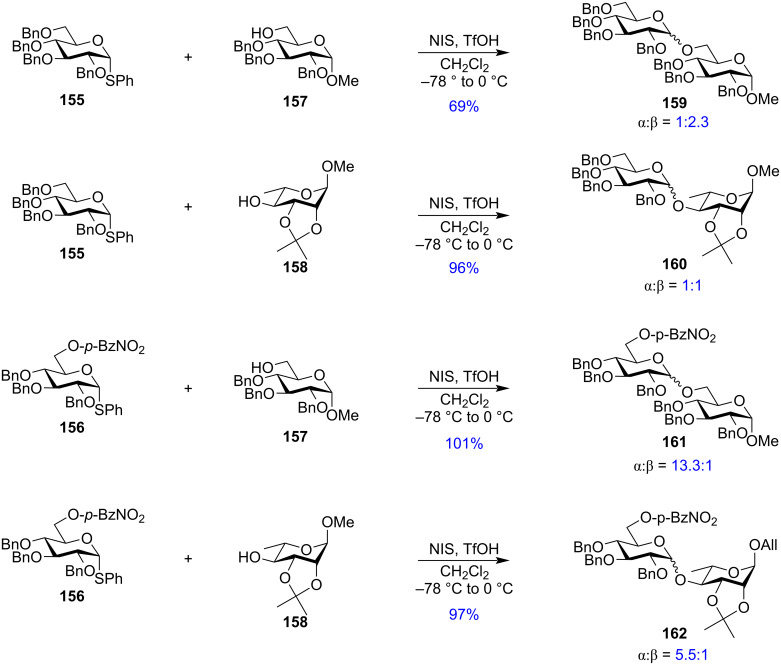
The role a C-6 *p*-nitrobenzoyl group on the stereochemistry of the glycosylated product [196].

The study by Boltje and Codée et al., however, could not find any suitable evidence depicting the role of the C-6 ester functionality on the stereochemistry of the glycoside formed in the glycosylation reaction by formation of any bridged intermediate. They assessed the effect of long-range participation of acyl groups by a combination of three approaches consisting of IRIS, CEL computations, and glycosylation reactions and stated that the remote participation follows the order: 3-Ac-Man >> 4-Ac-Gal > 3-Ac-Glu ≈ 3-Ac-Gal > 4-Ac-Glu > 4-Ac-Man ≈ 6-Ac-Glc/Gal/Man [186]. However, the work by Jensen et al. showed distinct changes in the stereochemistry of the glycosylated products by using a variety of ester groups as the C-6 temporary protection. The best 1,2-*cis* or α-selectivity was obtained using donor **156** having a *p*-nitrobenzoyl protecting group in the C-6 position. The group compared the stereo-outcome of the glycosylation reaction with donors having non-participating benzyl and participating ester groups in the C-6 position in the presence of both primary and secondary acceptor **157** and **158**, respectively (Scheme 27). The C-6 benzylated substrate yielded anomeric mixtures of **159** and **160**, while the presence of a C-6 remote *p*-nitrobenzoyl group significantly increased the percentage of the 1,2-*cis* anomer in **161** and **162** [196]. This observation although providing some evidence on the role of C-6 ester protection on stereodirecting the outcome of the glycosylation reactions, Jensen et al. could not find any direct evidence of any remote ester participation. They argued that probably the introduction of a strongly electron-withdrawing group like a nitrobenzoate perturbs the mechanistic pathway of the glycosylation toward a lesser dissociative S_N_1-type component as the oxocarbenium ion-like intermediate is destabilised by the electronic effect exerted by the *p*-nitrobenzyl group. Thus, the probability of distal participation was rejected.

**Scheme 28:**
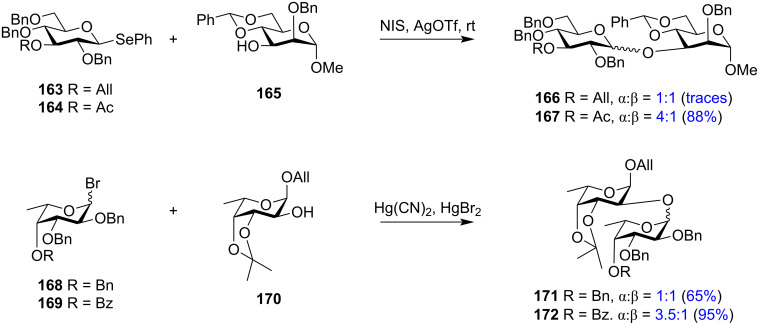
Difference in stereoselectivity obtained in glycosylation reactions by replacing non-participating groups with participating groups in the C-3 and C-4 position [198].

With the illustrated participation by remote acyl groups on the stereochemistry of the produced glycosides, similar ester protections like benzoyl, pivaloyl, or chloroacetyl also exhibited a comparable participating nature to form 1,2-*cis* glycosides. A recent report on the synthesis of the repeating unit of the pentasaccharide of the *O*-antigen of *A. baumanni* ATCC 17961 has been accomplished using the remote long-range participation of the levulinoyl group is an application of the role of distal ester groups in oligosaccharide synthesis [197]. Reported examples distinctly illustrating the role of the remote ester groups in increasing the ratio of 1,2-*cis* glycosides are shown in Scheme 28. The glucopyranosyl donor with an acetyl group **164** in remote C-3 position exhibited a 4 times higher 1,2-*cis* stereoselectivity than donor **163** with an allyl protection in the C-3 position [198]. Similarly, remote 4-*O*-benzoyl ester-protected ʟ-glycopyranoside donor **169** led to the formation of more 1,2-*cis* stereodirected product compared to the benzyl-protected donor **168** [199]. Both reports ascertain that remote participation by ester protecting groups as one of the many stereo-driving factors in glycosylation reactions.

**Scheme 29:**
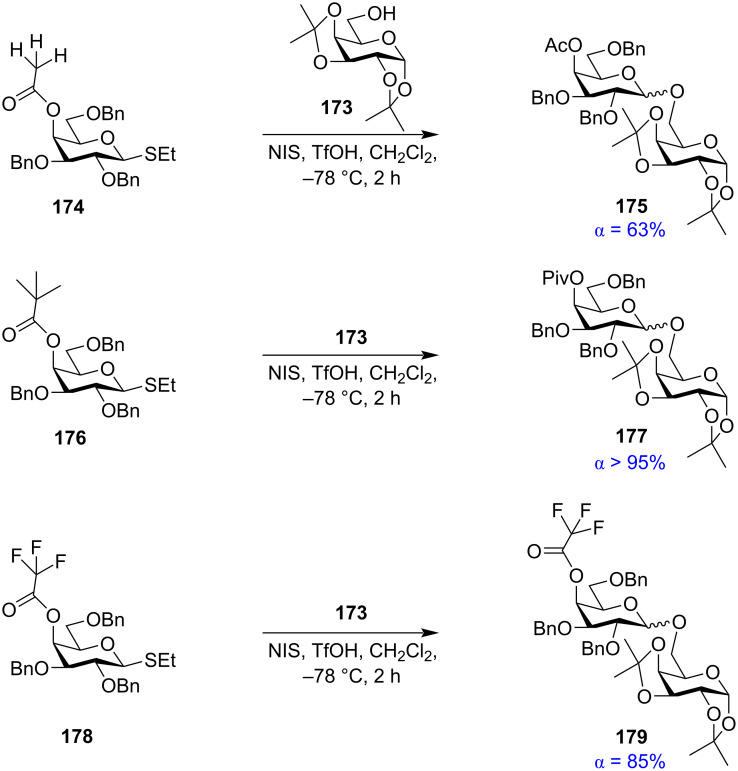
The role of electron-withdrawing and electron-donating substituents on the C-4 acetyl group in glycosylation reactions [199–200].

Very recently, Seeberger and co-workers studied the role of electron-donating and electron-withdrawing substituents on the acetyl groups and their contribution towards the remote participation in glycosylation reactions (Scheme 29) [200]. It was thereby observed that while 4-*O*-acetyl-protected galactopyranoside **174** on activation in the presence of the strong nucleophilic primary acceptor **173** produced 63% of the 1,2-*cis* glycoside **175**. The presence of more electron-donating substituents on the distal acetyl groups like a pivaloyl group in **176** increased the efficiency of remote participation thereby leading to almost exclusive (>95%) formation the 1,2-*cis* glycoside product **177**. Thus, the pivaloyl (Piv) group was seen to be an effective remote participating group. On the contrary, the presence of more electron-withdrawing substituents on the acetyl group like trifluorinated (TFA) derivative **178** produced reduced amounts (85%) of 1,2-*cis* glycosylated product **179**. However, counterintuitively, the α-selectivity for the 4-TFA protection was higher than the corresponding acetyl groups which significantly deviated from the expected results. Seeberger and group performed a set of experiments to infer that favourable formation of α-selective β-triflates played a role here and thereby the 4-TFA group was not involved in remote participation. Cryogenic infrared spectroscopy and density functional theory (DFT) investigations of glycosyl cations showed the formation and collaboration of the connecting dioxolenium, oxocarbenium, and rearranged intermediate structures. This study also helped in the design and synthesis of oligosaccharides in an automated oligosaccharide synthesis protocol [201].

Thus, despite all the evidences, Crich et al. argued against the concept of distal or remote group participation by the formation of a bicyclic oxocarbenium ion intermediate based on various factors [32–34]. The group provided various insights stating that formation of the glycosyl oxocarbenium ion from the activated covalent donor is highly unflavoured, since glycosylation is dominated by the multiplicity of the electron-withdrawing, destabilising C–O bonds. The rigid bicyclic system shown as the evidence towards distal protection also comes with greater entropy cost due to the change in the conformation of the sugar ring. However, the formation of a seven-membered bridged bicyclic dioxocarbenium ion induced by probable remote group participation was first elucidated by low temperature solvent-phase NMR spectroscopy by Crich and co-workers by the introduction of an additional methyl group at the C-4 position of the galactopyranoside donors under typical solution-phase glycosylation conditions [185,202].

**Scheme 30:**
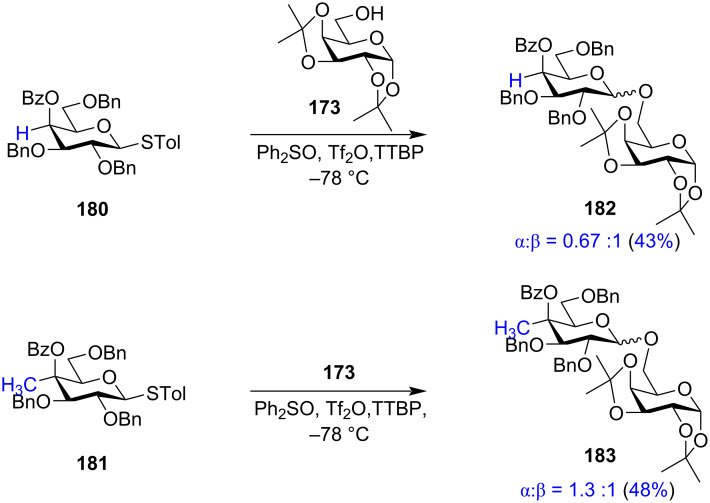
Effect of the introduction of a methyl group in the C-4 position on the glycosylation with more reactive primary nucleophiles [202].

Performing the glycosylation with primary acceptor **173** and comparing the stereodirecting property, it was observed that substituting the equatorial 4-H in **180** with a methyl group in **181** increased the selectivity towards the formation of the 1,2-*cis* anomer (Scheme 30). However, the difference in selectivity was not prominent with an acceptor with a secondary hydroxy group. Thus, although the formation of the bicyclic oxocarbenium ion was facilitated by the well-placed 4-CH_3_ substituent by destabilizing the ground-state ester conformation, the glycosylation results led Crich et al. to conclude that remote participation may not be the main reaction pathway for such glycosylation reactions. The minor difference in anomeric selectivity may be due to the inherent stereoselectivity of the donors with the acceptor by the formation of a loose, solvent-separated ion pair. In the context, Crich et al. suggested that the high 1,2-*cis* selectivity observed by Seeberger, Pagel and co-workers with the pivalate group in comparison to the corresponding acetyl group may be due to the increased electron density on the O-4 position induced by the presence of the *tert*-butyl group and the increased stabilisation of the positive charge on the anomeric carbon. They inferred that the stereochemical implication of the 4-*O*-acetyl group was mainly dependent on the concentration and stoichiometry of the reagents and thus remote group participation may be considered as a borderline phenomenon even in the most favourable cases [203].

**Figure 3:**
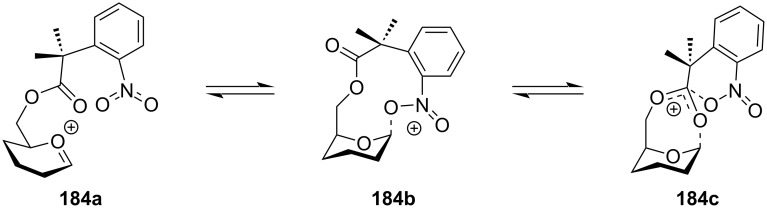
Remote group participation effect exhibited by the 2,2-dimethyl-2-(*o*-nitrophenyl)acetyl (DMNPA) protecting group installed at C-6 [204].

However, as the other side of the coin, in another illustration, the long-range remote participation effect of protecting groups present in the C-6 position was evidenced and experimentally proved by Codée et al. by using the 2,2-dimethyl-2-(*o*-nitrophenyl)acetyl (DMNPA) protecting group [204]. When mounted in the distant C-6 position, it distinctly stereodirected the glycoside product towards the formation of the more challenging 1,2-*cis* glycosides. However, the DMNPA group failed to produce any clear stereodirecting effects when installed in the remote C-3 or C-4 position. The authors provided experimental evidence supported by IRIS, that the formation of the C1, C6 dioxolenium ion is stabilised by the transfer of electron density of the aromatic nitro functionality (Figure 3). The formation of dioxolenium ion intermediate **184b** was attributed by the phenomenon of bringing the participating aryl nitro group in proximity to the anomeric carbon atom (**184c**) by virtue of the geminal dimethyl groups through the Thorpe–Ingold effect [205–206]. Herein, it was reported that the distal ester interacted with the anomeric carbon and the nitro-derivatised acyl group stabilised the interaction to produce the C1,C6 dioxolenium intermediate **184c**. The isolation of this stabilised ion intermediate thereby proved the interaction of the C-6 remote protecting group with the anomeric carbon to induce the directing stereochemistry of the glycosylation reactions. This has also been verified by various other experimental studies like X-ray crystallography and DFT calculations [207].

#### Carbonate protecting group

Crich et al. introduced the carbonate protecting group in glycosyl donors to effectively accomplish glycosylation reactions and control the stereochemistry of the produced glycosides. In conjunction with the 4,6-*O*-benzylidene protection, the 2,3-*O*-carbonate protection in mannopyranosides effectively led to the formation of the α-triflates [208], leading to subsequent S_N_2 attack by the nucleophile to form β-mannosides. However, NMR studies indicated the formation of the ^0^*H*_5_ half-chair conformation which was enforced by the conformational requirements of the cyclic 2,3-*O*-carbonate protection [209]. The 2,3-*O*-carbonate esters led to the formation of moderate to high β-selective mannosyl products via the formation of α-triflate intermediate. The fused system being *trans* in nature, hinders the formation of the oxocarbenium ion leading to α-triflate formation with subsequent S_N_2 attack of the acceptor from the β-face. However, the procedure required the preactivation of the donor molecule by BSP and triflic anhydride in CH_2_Cl_2_ at low temperature prior to the addition of the nucleophilic acceptor [210]. This method was specifically applicable for the formation of the synthetically challenging β-mannopyranosides and β-rhamnosides. The 4,6-di-*O*-acetyl-2,3-*O*-carbonate-protected thioglucoside and thiogalactoside donors gave high α-selectivity on being activated by catalytic amounts of Lewis acids like BF_3_·Et_2_O or stoichiometric amounts of Lewis acid additive like SnCl_4_ [211]. Similarly, 3,4-*O*-carbonate esters also gave high α-stereoselectivity to 2-deoxy sugars and 2,6-dideoxy sugars owing to its conformationally constraint feature [212]. Based on the same conformationally constrained donors, 2,3-*trans*-fused *o*-xylylene (Xyln) [213–214], 1,1,3,3-tetraisopropyldisiloxane-1,3-diyl (TIPDS) [215], and 2,3-naphthalenedimethyl [216] protecting group were used, all of which rendered excellent β-stereoselectivity. Herein, NMR and DFT simulation studies, revealed the glycosylation to proceed via a ^4^*H*_3_ conformational intermediate.

Thus, conformational torsional strain exerted by the different cyclic protecting groups and the disarming nature exhibited by them on the ring structure contribute widely in controlling the stereochemistry of the produced glycosides [217]. Moreover, modulating the stereoelectronic contributions owing to the position and stereochemistry of the remote protecting groups significantly helps in stereodirecting the glycosylation reaction in the preferred pathway.

Thus, by the introduction of the 2,3-*O*-carbonate group or *N*-acetyloxazolidinone protection, glycochemists showed the stereodirecting effect of the glycosyl donors which was accomplished, particularly, in the absence of any neighbouring group or any solvent participation, implementing the importance of the conformation factors. The role of the distal carbonate protection is, however, more dependent on the formation of the subsequent triflates and the rate of decomposition of the latter in the presence of moderately reactive nucleophiles. So, the effect of the cyclic protection may be basically demarcated as conformational influence instead of remote participation in the context of glycosylation reactions.

#### Other influences by remote protecting groups

**Scheme 31:**
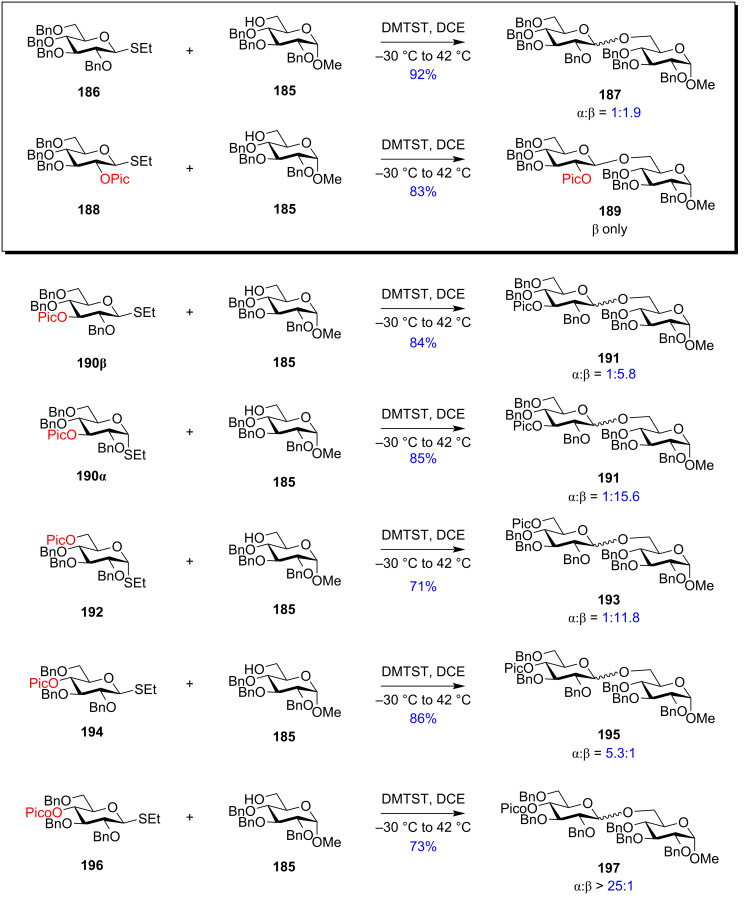
The different stereoselectivities obtained by Pic and Pico donors on being activated by DMTST.

**Hydrogen bond-mediated aglycon delivery-(HAD): picoloyl and picolinyl remote protecting groups:** While describing neighbouring group participation effects (Scheme 16), we have previously illustrated the use of pyridine-containing 2-*O*-picoloyl or picolinyl (Pic) groups as a vicinal protecting group with Pic-protected **188** giving the 1,2-*trans* glycosylated product **189** in good yield. It portrayed the use of ether-type vicinal protecting groups to afford 1,2-*trans* glycosides by the formation of a six-membered ring intermediate. The anomeric selectivity was significantly reduced with glycosyl donors without any Pic protection, such as compound **186**. Taking these two reactions as the control reactions, Demchenko et al. illustrated the use of picolinyl (Pic) groups or picoloyl (Pico) group as remote O-3, O-4 and O-6 protection to stereodirect the outcome of the glycosylation reactions (Scheme 31) [218]. As described earlier for the neighbouring Pic group, it was observed that the picoloyl group enabled the attack of the acceptor molecule from the side *trans* to the Pic vicinal protecting group implementing high 1,2-*trans*-selective glycosylation reactions. However, the authors observed completely different results when using the Pic or Pico group as remote groups at C-3 (**190α** and **190β**), or C-6 (**192**) position. It was seen that the glycosylated product was *syn* to the direction of the picolinoyl substituent instead of the anticipated *anti* product. In this regard, it was observed that the C-3-Pic-protected **190** and C-6-Pic-protected **192** glycosyl donors afforded higher 1,2-*trans* selectivity but the C-4 Pic-protected donor **194** led to higher yield of the 1,2-*cis*-selective glycoside product. Similarly, the Pico group when installed in the C-4 position **196** also showed an enhanced 1,2-*cis* selectivity. Demchenko et al. also investigated the role of the anomeric stereochemistry of the donor molecule by using the α-anomer of the 3-*O*-Pic-protected glycosyl donor **190α** which under similar activation conditions produced glycosides with an α:β ratio of 1:15.6 which is nearly a 3-fold enhanced β-selectivity when compared with the corresponding β-donor **190β** which produced glycosides with an α:β ratio of 1:5.8.

**Figure 4:**
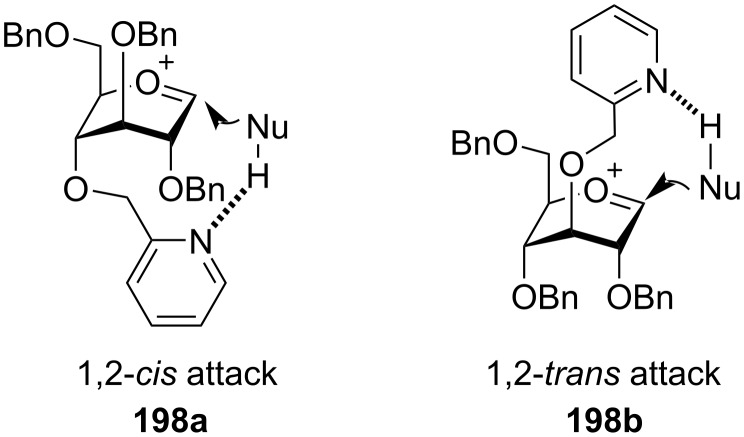
Hydrogen bond-mediated aglycon delivery (HAD) in glycosylation reactions for 1,2-*cis* **198a** and 1,2-*trans ***198b** attack.

Thus, to explain the *syn* stereoselectivity obtained by the contribution of the Pic and Pico groups, Demchenko et al. suggested the possibility of the reaction proceeding through hydrogen bond-mediated aglycon delivery (HAD) [219]. According to this postulate deduced by Crich and co-workers, the Pic and Pico groups acted as a potential H-bond acceptor from the attacking nucleophile. This intramolecular H-bond tethering between the pyridine moiety and the acceptor in turn restricts the attack of the acceptor nucleophile on the anomeric carbon to predominately occur from the *syn* position (Figure 4) [220]. The H-bond tethering **198a** and **198b** also helps to accelerate the glycosylation reaction by bringing the acceptor and donor components in close proximity thus facilitating the final deprotonation step.

**Scheme 32:**
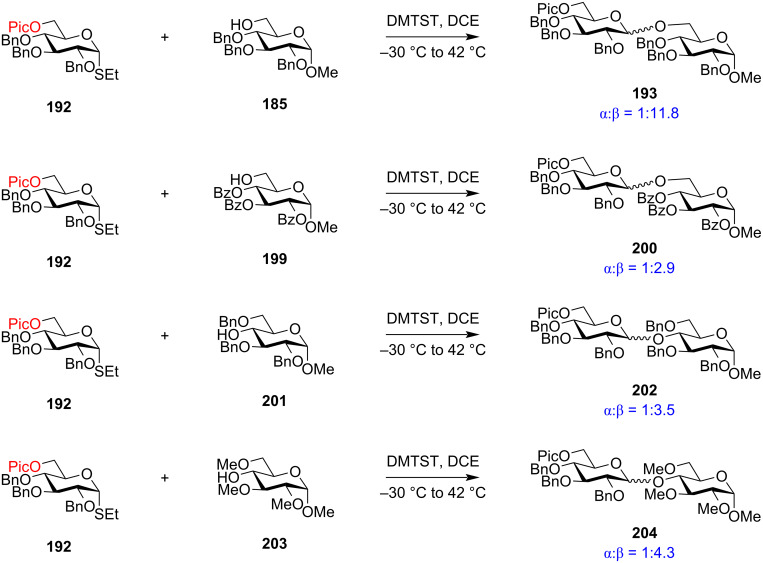
The role of different acceptor with 6-*O*-Pic-protected glycosyl donors.

The H-bond dependence on the reaction medium was illustrated by the fact that an almost 50 times dilution led to a much faster glycosylation and enhanced stereoselectivity. Increased dilution from 50 mM to 1 mM of the donor was found to increase the stereoselectivity due to a decreased probability of the non-stereoselective attack of the unbound nucleophiles. The result is indicative of the occurrence of the H-bond-mediated glycosyation. However, the obtained stereoselectivity was found to be significantly decreased with less reactive benzoylated acceptor **199** and sterically hindered secondary alcohol, **201** as acceptors [221] (Scheme 32). With more electron-withdrawing substituents in the glycosyl acceptor **199**, the nucleophilic hydroxy group becomes electron-poor and hence its hydrogen-bond-donating properties becomes considerably low. Thus, with electron-poor acceptors, the glycosylation fails to proceed through a hydrogen bond-mediated aglycon delivery pathway. The use of sterically hindered substituents as in secondary benzylated glycoside acceptor **201** also reduces the stereoselectivity. However, replacing the bulky benzyl groups with methyl counterparts in **203** leads to a slight improvement in stereoselectivity. Remote *O*-6-Pic protection also rendered promising β-selectivity with deoxy glycoside donors without any protective group in the C-2 position [222]. This method of using Pic and Pico protecting groups as remote protecting groups has been effectively and widely used for β-mannosides [223–224], β-rhamnosides [225–226], and α-sialic glycosides [227–230]. Similar the pyridine-derived 2-quinolinecarbonyl group was also demonstrated by Yang and co-workers as H-bond-acceptor to effectively direct the formation of β-arabinofuranosides [231].

Similar to the hydrogen bond-mediated aglycon delivery (HAD) mechanism demonstrated by the picoloyl and picolinyl groups, phosphine oxides have also been studied as possible remote stereodirecting groups for glycosylation reactions. Pyridine and its derivatives in the picoloyl moiety could interfere with the Lewis acid promoters. In contrast, phosphine oxides are generally more neutral and stronger H-acceptors [232]. So, Li et al. selected the 2-diphenylphosphinoylacetyl (DPPA) group as remote participating group which was observed to yield the glycoside product in high 1,2-*trans* selectivity [233]. It worked on the principle of HAD between the phosphine oxides and the incoming acceptor moiety. Control reactions blocking the possible H-bonding were also performed to confirm the mechanistic protocol. This protecting group has also been successfully implemented for target-oriented multistep oligosaccharide syntheses. Similarly, DPPA installed in *N*-phenyltrifluoroacetimidate donors was effectively used in glycosylation and played a dual participating role as a leaving group and as a remote protecting group in the C-6 position. Low temperature NMR studies showed 1,6-bridged bicyclic trifluorooxazepinium ions which in turn led to the efficient conversion to glycoside products with high 1,2-*cis* stereoselectivity [234].

Encouraged by the concept of hydrogen bond-mediated aglycon delivery, Demchenko et al. also reported the use of halobenzoyl groups in the C-6 position to stereodirect the outcome of the glycosylation reactions [235]. Whereas a 6-*O* fluorinated benzoyl group was expected to exhibit almost nil or extremely weak effects on the glycosylation reactions, studies with bromo- and chlorobenzoyl protecting groups were demonstrated to yield glycoside products with high 1,2-*cis* selectivity. However, Demchenko and co-workers were unable to portray any evidence confirming the involvement of HAD in the reaction protocol. The halobenzoyl groups, however, drastically altered the disarming nature of the benzoyl groups in the said glycosylation protocol thereby enhancing the reactivity of the glycosyl donor.

**Benzylidene acetal protecting groups:** The role of the acetal protection on the glycosyl donor in directing the stereochemistry of the products remains a debated topic of study. From different reports, it has been seen that acetal protection does not directly contribute to the stereochemistry of the products. However, it exhibits multiple conformational and electronic effects on the glycosyl donor. It has also been reported that 4,6-*O*-benzylidene acetal protecting groups also render a distinct but indirect remote effect on the sugar ring thereby contributing towards the formation of the α-triflate-functionalised mannosides which by subsequent attack by the incoming nucleophile led to the formation of the challenging β-mannose glycosides [38–39]. It was observed that the benzylidene protection locked the C-6 bond of the sugar ring in the *trans-gauche* (*tg*) conformation. This made the 6-CO bond to be antiperiplanar to the 5-CO bond. Thus, the electron-withdrawing nature of the benzylidene group is maximised making the donor electron-poor thereby disarming the nature of the glycosyl donor [36,236]. Thus, a less stable oxocarbenium intermediate is formed shifting the equilibria as far as possible toward the covalent triflate leading to the stabilisation of the α-triflates as the intermediate primarily. These triflates undergo S_N_2-like substitution by the attack of the strong nucleophile [237] to form β-mannosides. The formation of the intermediate *tg* conformation was verified using kinetic isotope studies [238]. The role of the disarming effect of benzylidene and its role on mannosylation reactions has been effectively and elaborately studied by Pedersen and co-workers who emphasised on the stereoelectronic locking of the tethered C-6 bond of the sugar ring [239].

**Scheme 33:**
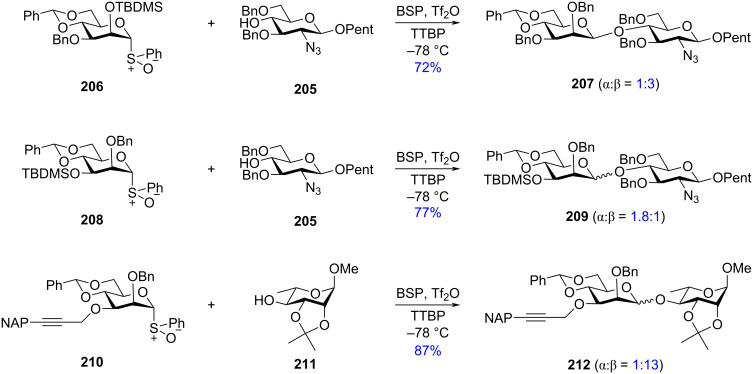
The role of the remote C-3 protection on various 4,6-*O*-benzylidene-protected mannosyl donors affecting the stereoselectivity of the produced mannoside.

However, Misra et al. showed that the benzylidene groups largely depended on the functionality of the C-3 position. The presence of ether groups like *p*-methoxybenzyl (PMB) or *O*-naphthylmethyl (NAP) in the C-3 position helps in the implementation of the reaction without any low temperature preactivation of the donor [240] and attain more β-selectivity. This was confirmed when the TBDMS group was used in the C-3 position of the mannosyl donor **208** which significantly reduced the stereoselectivity of the reaction to produce slight excess of the α-mannoside **209** as the major product [241] (Scheme 33). Similarly, 1-naphthylpropargyl protection in the C-3 position of 4,6-benzylidene-protected mannoside donor **210** significantly boosted the β-selectivity of the glycoside product **212** [195]. The use of functionalised benzylidene groups for stereodirecting the β-glycoside product was exhibited by Crich and co-workers. They used 4,6-*O*-[(*R*)-(2-(2-iodophenyl)ethylthiocarbonyl)benzylidene] group as the remote protecting group to accomplish the formation of β-rhamnoside derivatives [242]. However, the benzylidene protection gave 1,2-*cis* products for gluco- and galactopyranosides, rendering the role of benzylidene protection restricted only to the formation of β-mannosides.

**Scheme 34:**
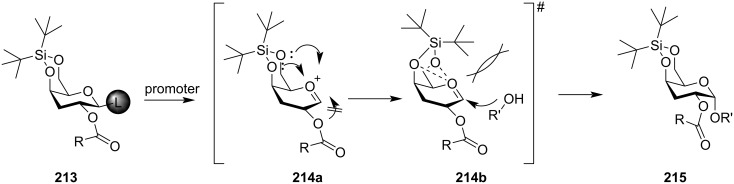
The dual contribution of the DTBS group in glycosylation reactions [246–247].

In sync with the benzylidene protecting group, the di-*tert*-butylsilylene (DTBS) protecting group tethering the 4-*O* and 6-*O* position has also been extensively used for the formation of stereoselective 1,2-*cis* glycopyranoside products [243–244]. It has been observed through X-ray crystallography that the sugar ring attains the near half-chair conformation to facilitate the formation of the required stereo- and regioselective products. This protecting group has particularly been extensively used to attain the 1,2-*cis* configuration with galactopyranoside donors [245]. The DTBS protecting group not only masks the C-4 and C-6 hydroxy groups but also increases the ‘space electron-donation’ thereby stabilising the oxocarbenium intermediate **214a**. It even neutralises the role of any participating group in the C-2 vicinal position (Scheme 34) [243]. The di-*tert*-butylsilylene group significantly increases the electronegativity of the C-4 oxygen atom. Owing to the high electronegative substituent in the equatorial C-4 position the intermediate oxocarbenium ion gets stabilised thereby not allowing any participation from the groups of the vicinal C-2 position. Moreover, the steric bulk of the silyl acetal protection **214b** also renders a ‘dual’ effect on the sugar ring enabling the formation of 1,2-*cis* stereoselective glycoside products **215**. Thus, DTBS-assisted stereoselective 1,2-*cis* galactosylations have been widely implemented for the multistep oligosaccharide syntheses [246–247].

### Neighbouring group participation (NGP) vs remote group participation (RGP)

In glycosylation reactions, the role of protecting groups is highly important for modulating and directing the stereochemistry of the produced glycosides. As highlighted in the previous sections, the protecting groups neighbouring the anomeric position exhibit high stereodirecting properties to form 1,2-*trans* glycosides effectively. The ester protecting groups and their derivatives interact with the anomeric carbon to form the dioxolenium ion which leads to the formation of the required glycoside. The ether-type and chiral auxiliary protecting groups also contribute effectively towards the formation of highly stereoselective glycosides. The phenomenon of neighbouring group participation by the vicinal protecting groups is widely accepted in synthetic glycochemistry for oligosaccharide synthesis to achieve effective stereochemical outputs.

In comparison, the concept of remote participation is a highly debated topic and much study is still going on to ascertain the phenomenon in glycosylation reactions. Remote participation of the distal protecting groups, however, shows a possibility of controlling the stereochemistry of the produced glycosides to attain 1,2-*cis* or 1,2-*trans* isomers. Long-range association with the remote protecting groups, however, is highly dependent on several stereoelectronic parameters. The remote participation deals with the change of conformation of the sugar ring enabling the interaction of the participating groups from the distal positions with the anomeric carbon. Although there is a possibility of stabilisation of the naked oxocarbenium ion, the formation and isolation of the oxocarbenium ion in solution phase is against various stereoelectronic parameters, which have led various glycochemists to question its authenticity. The association of the solvent ion pair is indicative of the reaction to proceed via an S_N_2-like mechanism and less of an S_N_1 component. However, the oxocarbenium intermediate, indeed, has been successfully characterised by several computational methods, CREST NMR and IRIS. The concepts still require further study to ascertain the participating nature of the ester groups in distal positions. It is also observed that in the glycosylation reaction, a less stable oxocarbenium ion shifts the equilibrium to stable covalent glycosyl triflate as intermediate and the reaction shifts towards more S_N_2-like component by the attack of moderate to strong nucleophiles.

Remote protecting groups often participate in hydrogen bond-mediated aglycon delivery (HAD) via specific protecting groups like picoloyl, phosphine oxides, etc. The HAD mechanistic protocol is particularly helpful for the development of synthetically challenging β-mannoside, β-rhamnoside, or α-sialoside derivatives. Pic groups have also been rendered to be an effective stereodirecting protecting group in deoxy glycosides with no protecting groups in the C-2 position.

However, the role of the remote participating groups on the stereochemistry of the produced glycoside is much less prominent when compared with the vicinal protecting group participation. Complete stereocontrol of the glycosylation reactions depends on a variety of other factors apart from remote participation of distant protecting groups. The nature of the incoming nucleophiles also plays a role in stereoselectivity of glycosylation reactions. It has been observed that strong nucleophiles likely proceed through an S_N_2 mechanism enabling inversion of configuration. Varying the nature of the incoming nucleophile acceptor molecule often changes the stereochemistry of the obtained product starting from the same glycopyranoside donor with same participating groups in the distant positions. Higher stereocontrol is obtained with more reactive primary alcohols while electron-withdrawing and less reactive secondary acceptors yield much reduced stereocontrol [185,196,202,221]. The incorporation of the role of nucleophile, significantly reduces the importance of the role of distal participation of the protecting groups in the C-3, C-4, and C-6 positions.

Thus, we can safely conclude that completely directing the stereochemistry of the produced glycoside bond to either 1,2-*cis* or 1,2-*trans* solely based on the participation of the distant protecting groups of the C-3, C-4, or C-6 position is not possible. In a very recent article, Gu, Tang, Cai and co-workers chose to use the term acyl group ‘direction’ instead of ‘participation’ [248]. They have shown that the acyl groups help in both participation as well as H-bond interaction in a weakly nucleophilic environment. However, the roles are contradictory and it is essential to reduce the H-bond interaction to get an optimal participatory effect of the acyl groups.

Apart from this, remote group participation brings with it a variety of limitations in its mechanistic protocol. Remote group participation could not completely explain the difference in the stereodirecting property of the acyl group present in the same position of different sugar residues [249]. The 3-*O*-acyl group gives complete 1,2-*cis* stereoselectivity to fucopyranosyl donors while imparts a very little effect for glucopyranosyl donors [182]. While the 3-*O*-acetyl group rendered increased α-selectivity in benzylidene-protected mannopyranosyl donors as explained in previous sections, there also have been reports showing 4,6-*O*-benzylidene hindering the possible participating assistance of the remote acyl groups increasing β-selectivity. The mechanistic studies to analyse and interpret the deviations from the expected mode of participation of the distal protecting groups have been studied and newer concepts have been demonstrated by different groups. Further studies are being widely conducted across the globe to standardise and delineate the contribution of the remote participating groups on glycosylation mechanism. Many further studies are still required to regularise this concept of remote participation and implement its applicability in multistep oligosaccharide syntheses.

## Conclusion

Proper protecting group manipulation is highly essential in carbohydrate chemistry. Neighbouring group participation of temporary protections in the C-2 position, vicinal to the anomeric carbon, is highly versatile enabling the formation of 1,2-*trans* glycosides primarily. Electron-withdrawing protecting groups in the C-2 position, however, have been attributed to disarming and reduce the reactivity of the glycosyl donors affecting the versatility of the glycosylation reactions. In this context, there have been reports regarding the implementation of auxiliary groups participating with the anomeric carbon forming bicyclic intermediates, in turn, affecting the stereochemistry of the produced glycosides. This stereoselectivity of the glycosylation reactions can also be enhanced by using similar functional groups in the remote positions, which may act as additional factors and may affect the stereocontrol of the reactions. There are no specific remote functional groups in oligosaccharide synthesis, wherein the same acetyl, benzoyl, picolinyl groups can act as both vicinal and distal protecting groups exhibiting different roles on the glycosylation reactions.

Thus, we can safely conclude that extensive stereocontrol in oligosaccharide synthesis depends on a variety of factors like solvent system, temperature control, nature of the acceptor molecule, etc., coupled with the participation offered by both neighbouring and remote protecting groups. Thus, neighbouring group participation and remote group participation are mutually responsible for attaining the desired product and both mechanistic protocols should be carefully analysed and the synthetic protocols should be designed accordingly. Lastly, oligosaccharide synthesis is still one of the most intriguing and challenging areas of research for organic chemists as a whole and glycochemists in particular. As Hans Paulsen said [250], ‘Although we have now learned to synthesise oligosaccharides, it should be emphasised that each oligosaccharide synthesis remains an independent problem, whose resolution requires considerable systematic research and a good deal of know-how. There is no universal reaction condition for oligosaccharide synthesis.’ Neighbouring group participation (NGP) and remote group participation (RGP) are just an effort to regularise the glycosylation techniques and provide glycochemists an extensive protocol to design their multistep oligosaccharide synthesis in an effective way.

## Data Availability

Data sharing is not applicable as no new data was generated or analyzed in this study.
